# Molecular pathology of endocrine gland tumors: genetic alterations and clinicopathologic relevance

**DOI:** 10.1007/s00428-023-03713-4

**Published:** 2023-12-18

**Authors:** Antonio De Leo, Martina Ruscelli, Thais Maloberti, Sara Coluccelli, Andrea Repaci, Dario de Biase, Giovanni Tallini

**Affiliations:** 1 Department of Medical and Surgical Sciences (DIMEC), University of Bologna, 40138 Bologna, Italy; 2 Solid Tumor Molecular Pathology Laboratory, IRCCS Azienda Ospedaliero-Universitaria di Bologna, 40138 Bologna, Italy; 3 Division of Endocrinology and Diabetes Prevention and Care, IRCCS Azienda Ospedaliero-Universitaria di Bologna, 40138 Bologna, Italy; 4 Department of Pharmacy and Biotechnology (FaBit), University of Bologna, 40126 Bologna, Italy

**Keywords:** Molecular pathology, Endocrine gland tumors, PitNET, Thyroid tumors, Parathyroid tumors, Adrenal cortical tumors, Paraganglionic tumors

## Abstract

Tumors of the endocrine glands are common. Knowledge of their molecular pathology has greatly advanced in the recent past. This review covers the main molecular alterations of tumors of the anterior pituitary, thyroid and parathyroid glands, adrenal cortex, and adrenal medulla and paraganglia. All endocrine gland tumors enjoy a robust correlation between genotype and phenotype. High-throughput molecular analysis demonstrates that endocrine gland tumors can be grouped into molecular groups that are relevant from both pathologic and clinical point of views. In this review, genetic alterations have been discussed and tabulated with respect to their molecular pathogenetic role and clinicopathologic implications, addressing the use of molecular biomarkers for the purpose of diagnosis and prognosis and predicting response to molecular therapy. Hereditary conditions that play a key role in determining predisposition to many types of endocrine tumors are also discussed.

## Introduction

The endocrine system includes several organs all devoted to the physiologic role of maintaining homeostasis and mediating medium- to long-term reactions of the human body to adapt it to external modifications. Tumors of the main endocrine glands, anterior pituitary (adenohypophysis), thyroid and parathyroid glands, adrenal cortex, adrenal medulla, and paraganglia are the object of this review. Tumors of the diffuse neuroendocrine system are not included, excellent reviews have comprehensively covered the topic [1]. A variety of tumors and nodules develop in endocrine glands, with different pathologic features and clinical behavior. Some are very common. Indeed, small indolent foci of papillary carcinoma are found in ~ 35% of well-sampled thyroid glands at autopsy. Others, like parathyroid carcinoma, are very aggressive, but also very rare. A significant minority of endocrine gland tumors develop in the context of inherited syndromes and paraganglionic tumors of the adrenal medulla and paraganglia have the highest degree of hereditability among human neoplasms. Table 1 summarizes inherited syndromes of endocrine tumors [2–18]. Our knowledge of the molecular pathogenesis of endocrine gland tumors has exploded in the recent past due to the application of high-throughput molecular analysis. These studies show a remarkable correlation between genotype and histologic phenotype. They are also allowing us to refine risk stratification for prognostic purposes, as well as providing targets for molecular therapy in the case of aggressive endocrine gland carcinomas. The purpose of this review is to summarize the principal findings and innovations in the field of endocrine gland tumors in order to provide a state-of-the-art outline of molecular alterations and their clinicopathologic relevance.

**Table 1:** Hereditary syndromes associated with endocrine tumors

Gene(s)	Germline molecular alteration	Syndrome	MIM number	Inheritance transmission	Phenotype	Reference(s)
*AIP*	Missense mutations	Familial isolated pituitary adenoma (FIPA)	102200	Autosomal dominant (variable penetrance)	Endocrine tumors: isolated pituitary neuroendocrine tumor (PitNET, pituitary adenoma) (usually somatotroph	Beckers et al. [2]
*CDC73*	Inactivating mutations or deletions	Hyperparathyroidism jaw tumor (HPTJT) syndrome	145001	Autosomal dominant	Endocrine tumors: parathyroid tumor Non-endocrine tumors: jaw, renal and uterine tumors (uncommon) Clinical features: primary hyperparathyroidism	Masi et al. [3]; Sarquis et al. [4]
*CDKN1B*	Missense mutations, frameshift, and nonsense alterations in 5′ UTR	Multiple endocrine neoplasia type 4	610755	Autosomal dominant	Endocrine tumors: parathyroid, pancreatic, PitNET Clinical features: hyperparathyroidism	Alrezk et al. [5]; Frederiksen et al. [6]
*DICER1*	Inactivating frameshift, nonsense, splice-site mutations or deletions	DICER1 syndrome	138800 601200 180295	Autosomal dominant	Endocrine tumors: multiple benign and malignant thyroid nodules/tumors and pituitary blastoma Non-endocrine tumors, including: pleuropulmonary blastoma (PPB), cystic nephroma (CN), embryonal rhabdomyosarcoma (ERMS) of the uterine cervix, ovarian Sertoli-Leydig cell tumor (SLCT)	de Kock et al. [7]
*MAX*	Nonsense and missense variants, large deletions	Multiple endocrine neoplasia type 5	171300	Autosomal dominant	Endocrine tumors: nodules/tumors at multiple sites, including adrenal cortex, adenohypophysis, pancreas, and parathyroid Non-endocrine tumors: renal cell carcinoma and oncocytoma, breast, lung, and endometrial carcinomas	Burnichon et al. [8]; Korpershoek et al. [15]; Cascoń and Robledo [9]
*MEN1*	Premature truncation, frameshift, nonsense mutations, and large deletions (including intronic areas damage with splice site defects)	Multiple endocrine neoplasia type 1	131100	Autosomal dominant	Endocrine tumors: nodules/tumors at multiple sites, including parathyroid, adenohypophysis, pancreas and duodenum, adrenal cortex Clinical features: hyperparathyroidism, the most common manifestation	Brandi et al. [10]
*NF1*	Heterozygous variants	Neurofibromatosis type 1	162200	Autosomal dominant	Endocrine tumors: pheochromocytoma Non-endocrine tumors: neurofibromas at multiple sites, gliomas, malignant peripheral nerve sheath tumor, gastrointestinal NET Clinical features: pigmentation (including choroidal freckling), Lisch nodules	Ferner et al. [11]
*PRKAR1A* *PDE8B* *PDE11A*	Inactivating mutations, typically affecting *PRKAR1A* (sometimes large *PRKAR1A* deletions)	Carney Complex	160980	Autosomal dominant	Endocrine tumors: primary pigmented nodular adrenocortical disease (PPNAD) Non-endocrine tumors: myxoma and pigmented schwannoma Clinical features: spotty skin pigmentation	Stratakis [12]
*RET*	MEN 2A -Activating mutation (usually codons 609, 611, 618, 620 or 634)	Multiple endocrine neoplasia type 2A	171400	Autosomal dominant	Endocrine tumors: medullary thyroid carcinoma, pheochromocytoma, parathyroid nodules/tumors, or isolated medullary carcinoma (condition formerly known as Familial medullary carcinoma)	Wells et al. [13]; Moline and Eng [14]
	MEN 2B -Activating mutation (usually M918T)	Multiple endocrine neoplasia type 2B	162300	Autosomal dominant	Endocrine tumors: early onset, highly aggressive medullary carcinoma, pheochromocytoma, oral and intestinal neuromas Clinical features: marfanoid habitus	
*SDHA* *SDHB* *SDHC* *SDHD* *SDHAF2*	Missense mutations involving genes of one of the Succinate dehydratase subunits	Succinate dehydratase (SDH) deficient neoplasia	606864 601650 614165 605373 606764 168000 171300	Autosomal dominant	Endocrine tumors: paraganglionic tumors (paraganglioma/pheochromocytoma, PPGL) Non-endocrine tumors: gastrointestinal stroma tumors and SDH-deficient renal carcinoma	Korpershoek et al. [15]; Gill [16]
*VHL*	Missense mutations, deletions or truncating mutations (less frequently)	Von Hippel Lindau syndrome	193300	Autosomal dominant	Endocrine tumors: PPGL, pancreatic NET Non-endocrine tumors: angiomas and hemangioblastomas, clear cell renal cell carcinoma, serous pancreatic cystadenoma, tumors of endolymphatic sac, epididymis, broad ligament and mesosalpinx	Lubensky et al. [17]; Salama et al. [18]

*MIM* Mendelian Inheritance in Man

## PitNET (pituitary adenoma): molecular pathology and correlation with clinicopathologic features

**Fig. 1:**
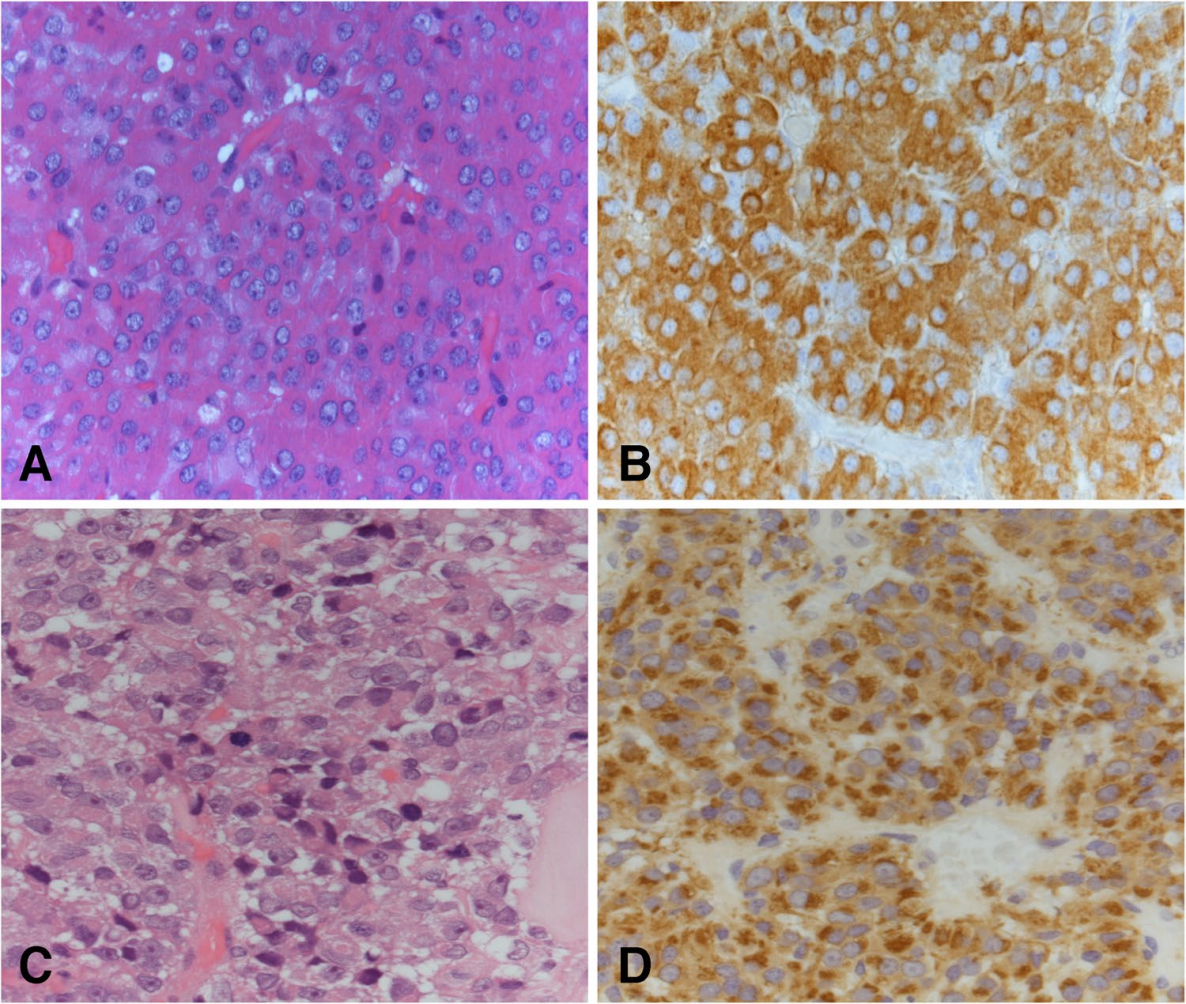
PitNET (pituitary adenoma). GH-producing densely granulated PitNET (**A**, hematoxylin and eosin; **B**, GH immunohistochemistry): *GNAS1* mutations occur in a subset of GH-producing pituitary adenoma, more frequently in densely granulated tumors. PRL-producing sparsely granulated PitNET with Golgi pattern PRL staining, the tumor was mitotically active and eventually metastasized to the brainstem and cerebellum (**C**, hematoxylin and eosin; **D**, PRL immunohistochemistry): the molecular pathogenesis of metastatic PitNET is still unclear

Pituitary adenomas, now termed pituitary neuroendocrine tumors (PitNET), originate from the six neuroendocrine hormone-secreting cell types derived from three main lineages: SF1-lineage gonadotrophs, TPIT-lineage corticotrophs, PIT1-lineage somatotrophs, lactotrophs, mammosomatotrophs, and thyrotrophs. Examples of PitNET are illustrated in Fig. 1 [2–25]. Table 2 is a summary of the main genes mutated in PitNET [19–24, 26].

**Table 2:** Somatic genetic alterations of PitNET (Pituitary adenoma) and their clinicopathologic relevance

Gene(s)	Molecular pathology	PitNET (Pituitary Adenoma) type(s)	Clinicopathologic implications	Reference(s)
*ATRX*	Nonsense mutations, frameshift indels, and rarely intragenic deletions inactivate ATRX, promoting telomere instability and cells immortality	Corticotroph	Aggressive, recurrent, metastatic disease	[27]
*GHR*	Hetherozygous substitution of a single codon of *GHR* exon 4 affects the stability and/or processing of the GH-receptor	Sparsely granulated somatotroph	Therapeutic implications: response to GH-antagonists	[28–30]
*GNAS*	Missense mutations at Arg201 or Gln227 result in constitutive activity of Gs-alpha subunit (Gsα) which increases c-AMP levels	Densely granulated somatolactotroph	Florid acromegaly Small tumors Hypointensity on MRI T2 sequences Therapeutic implications: response to somatostatin analogues	[28, 31]
*USP48*	Missense mutations of *USP48*, including M415I/V substitution, result in enhanced proopiomelanocortin (POMC) gene’s promoter activity	Densely granulated USP8- wild-type corticotroph	Therapeutic implications: response to Pasireotide (due to high SSTR5 expression)	[32]
*USP8*	Missense mutations, primarily in exon 14, of the Ubiquitin Specific Peptidase gene determine ACTH degradation and increased deubiquitination of EGFR	Densely granulated corticotroph	Florid Cushing disease Small tumors Therapeutic implications: response to Pasireotide (due to high SSTR5 expression)	[33–37]

Although the majority of PitNET lacks known recurrent driver mutations, several — sometimes involving hormone synthesis pathways — have been identified in subsets of sporadic tumors. In addition, a small percentage of PitNET affects patients with inherited predisposition due to germline genetic alterations (Table 1). In some instances, these alterations can be inferred by immunohistochemistry but need to be confirmed by sequence analysis. An example is the immunohistochemical loss of Menin in the tumors of patients with MEN1 syndrome [38–40]. To date, morphologic classification, together with clinical and radiologic data, continues to be the best predictor of patient prognosis and therapy response. The relevance of genetic profiling for the diagnostic process is still being defined [19–25]. Epigenetic alterations, particularly those connected to chromatin remodeling and cell cycle regulation [25], play a major role in the development of PitNET, independently of the hormone-producing phenotype of the tumor. Genes frequently dysregulated by epigenetic modifications include the following: *CDKN2A*, *RB1*, *DAPK1*, *GADD45G*, *THBS1*, *RASSF1A*, *FGFR2*, *MGMT*, *CASP8*, *TP73*, *HMGA1*, *HMGA2* [41]. In general, chromosomal abnormalities do not correlate with prognosis but are more common in hormone-producing tumors compared with those not associated with hormone production (silent PitNET) [23].

Activating *GNAS* mutations have been reported in 40–60% of sporadic densely granulated somatotroph PitNET and are present as a mosaic in the 10–15% of patients with McCune–Albright syndrome that have excess GH, usually due to GH-producing cell hyperplasia and less commonly to a GH-producing PitNET [42]. Mammosomatotrope PitNET also exhibits *GNAS*-related alterations [23]. Conversely, in sparsely granulated somatotroph tumors, somatic mutations of the GH receptor (*GHR*) altering GH autoregulation and STAT signaling have been reported [20, 25].

In corticotroph PitNET, *ATRX* mutations correlate with aggressive biological behavior and distant metastasis [43]. Densely granulated biochemically functioning corticotroph tumors harbor *USP8* [44], *USP48*, and less frequently *BRAF* p.V600E mutations [22]. The role of these changes and their potential therapeutic implications are still controversial [24].

The distinctive molecular signature of lactotroph PitNET includes epigenomic alterations such as high expression of MYC targets and dopamine receptor D2 (*DRD2*) [23]. However, the *SF3B1* p.R625H hotspot mutation has been recently discovered in some lactotroph tumors characterized by high prolactin levels and short progression-free survival [45]. Furthermore, somatic *SDHA* mutations and *SDHD* loss of heterozygosity have been reported in rare spontaneous PRL-producing macrotumors [16, 46].

The molecular pathogenesis of metastatic PitNETs is still unclear, due to the rarity of these tumors. *ATRX* [19, 47] and *PTEN* [43] mutations have all been reported in some metastatic PitNETs.

## Thyroid tumors: molecular pathology and correlation with clinicopathologic features

Tumors of the thyroid gland enjoy a remarkable correlation between histologic phenotype and genotype. This correlation has contributed to refining the current classification scheme. The vast majority of tumors arising in the thyroid are of follicular cell derivation, most are benign, and when endowed with malignant potential, usually follow a very favorable clinical course. This generally favorable course is due to the first effective form of a molecularly targeted therapy, radioactive iodide treatment [48]. A small proportion of tumors are neuroendocrine, originating from parafollicular cells (C-cells). Since they always have malignant potential, they are classified as medullary carcinoma, which represents ~ 3–5% of all carcinomas of the thyroid gland. Up to 25% of medullary carcinoma is inherited in the context of MEN syndromes (Table 1).

Based on clinical outcome, malignant tumors of follicular cells are broadly divided into three groups: those that have a favorable prognosis, anaplastic (undifferentiated) thyroid carcinoma characterized by a very poor prognosis, and a third group of tumors that have intermediate prognosis. While tumors in the first group are histologically well differentiated with clearly defined papillary or follicular architecture or are composed of clearly recognizable oncocytic cells, tumors with very poor prognosis are undifferentiated (i.e., anaplastic). Tumors in the group with intermediate prognosis are often poorly differentiated but may also retain conventional histologic differentiation (papillary, follicular, oncocytic). Under the microscope, they have in common with the prognostically favorable tumor group at least some degree of histologic differentiation, while they share with anaplastic carcinoma high-grade features, i.e., the presence of high mitotic activity and/or tumor necrosis. This classification scheme for thyroid carcinoma of follicular cells based on prognosis is clinically relevant and has been endorsed by the latest 5th edition of the World Health Organization (WHO) scheme (Table 3). The group of tumors that are well differentiated is in turn histologically divided into three subgroups. The first subgroup is composed of tumors that are follicular patterned, which include follicular adenoma and follicular carcinoma (follicular carcinoma when there is the invasion of tumor capsule or of blood vessels), as well as tumors of the encapsulated follicular variant papillary carcinoma family: encapsulated follicular variant papillary carcinoma when there is the invasion of tumor capsule or of blood vessels, and NIFTP (non-invasive follicular thyroid neoplasm with papillary-like nuclear features) when no invasion can be identified [49]. These tumors have a *RAS*-like molecular signature following the 2014 TCGA molecular classification scheme [50] as discussed in the next paragraph. The second subgroup is that of conventional (i.e., not encapsulated follicular variant type) papillary carcinoma, characterized by the well-known alterations of nuclear morphology (nuclear clearing, irregular contours of the nuclear membrane, grooves, and pseudoinclusions) [49]. These tumors are characterized by infiltrative growth and typically make papillae, although sometimes they can have less typical features, such as infiltrative follicular or solid/trabecular growth, or other less common features that characterize the numerous papillary carcinoma subtypes [51]. These tumors have a *BRAF* p.V600E-like molecular signature following the 2014 TCGA molecular classification scheme [50], as discussed in the next paragraph. The third subgroup is that in which tumor cells are oncocytic and lack the nuclear alterations of papillary carcinoma. These tumors are characterized by homoplasmic mtDNA mutations [52] associated with dramatic DNA copy-number alterations with widespread loss of heterozygosity [53], as discussed in the next paragraph.

**Table 3:** Prognostically relevant classification of tumors of follicular cell origin

Outcome	Proliferative grading (mitoses and/or tumor necrosis)	Histologic differentiation (growth pattern)	Histotype
Favorable	Low	Present, good (papillae, follicles)	Papillary carcinoma
			Follicular carcinoma
			Oncocytic carcinoma
Intermediate	High		Differentiated high grade thyroid carcinoma (papillary, follicular, oncocytic)
		Present, poor (solid/trabecular/insular patterns)	Poorly differentiated thyroid carcinoma
Poor		Absent (u(ndifferentiated growth)	Anaplastic thyroid carcinoma

Modified from Table #28677, Prognostic risk-based classification of follicular cell derived carcinomas of the thyroid gland, in WHO Classification of Tumours Editorial Board: Endocrine and Neuroendocrine tumours, vol. 8. 5th edn. (International Agency for Research on Cancer, Lyon, France, 2022) https://tumourclassification.iarc.who.int]

Medullary thyroid carcinoma is the primary neuroendocrine tumor of the thyroid gland. In spite of the remarkable variability of cell morphology and growth patterns (none of which is prognostically relevant), the only subtype recognized by the current WHO 5th edition is the medullary microcarcinoma, i.e., a tumor measuring less than 10 mm (or less than 5 mm according to some authors) scheme. The WHO 5th edition emphasizes the importance of proliferative grading for medullary carcinoma, following the International Medullary Thyroid Carcinoma Grading System (IMTCGS) [54]. The IMTCGS, based on the evaluation of mitotic count and tumor necrosis (Table 4), is in line with the classification framework of neuroendocrine neoplasms [55].

**Table 4:** International Medullary Thyroid Carcinoma Grading System (IMTCGS)

IMTCGS grading	Tumor necrosis	Cell proliferation	
		Mitoses	Ki67 index
Low grade	No	< 5 mitoses/2 mm^2^	< 5%
High grade	At least one of the following: ≥ 5 mitoses/2 mm^2^, Ki67 ≥ 5%, tumor necrosis		

Modified from Xu B et al. International Medullary Thyroid Carcinoma Grading System: A Validated Grading System for Medullary Thyroid Carcinoma. J Clin Oncol. 2022 Jan 1;40(1):96–104. 10.1200/JCO.21.01329. Epub 2021 Nov 3. PMID: 34731032; PMCID: PMC8683221

The molecular landscape of thyroid tumors, particularly that of follicular cell derivation, has come into focus also thanks to next-generation sequencing and other high-throughput methods. One of the forces driving these studies has been the need to identify genomic alterations that can be targeted by pathway-specific molecular drugs in aggressive carcinomas that do not respond to conventional radioiodine therapy [54, 56–58]. Table 5 is a summary of the main genes involved in thyroid tumor development and progression and of their clinicopathologic relevance. Overall, results are very consistent and converge on several important points:

**Table 5:** Genetic alterations of thyroid tumors and their clinicopathologic relevance

Gene(s)	Molecular pathology	Thyroid tumor type	Clinicopathologic implications	Reference(s)
*ALK*	ALK is a transmembrane receptor tyrosine kinase involved in the development of the central and peripheral nervous systems. It becomes constitutively expressed in follicular cells as a consequence of ALK rearrangements, the most common involving STRN and EML4	Papillary carcinoma (< 5%), classic (typically follicular predominant growth pattern), or diffuse sclerosing High-grade non-anaplastic carcinoma of follicular cells, typically poorly differentiated thyroid carcinoma (< 5%) Anaplastic carcinoma (< 5%)	*ALK* fusion more common in papillary carcinoma from children/young adults Therapeutic implications: patients with *ALK*-rearranged tumors respond to targeted therapy with *ALK* inhibitors	Kelly et al. [59]; Chou et al. [60]; Godbert et al. [61]; Xu et al. [62]; Xu et al. [63]
*BRAF*	BRAF is a serine/threonine kinase of the MAPK signaling pathway that regulates cell differentiation, proliferation, and survival Activating mutations Hotspot mutations in exon 15; the most common substitution (95% of cases) is c.1799 T > A (p.V600E). *BRAF* p.V600E is the prototype of the *BRAF* p.V600E-like mutated tumor group (2014 TCGA molecular classification scheme) Other mutations include c.1801A > G (p.K601E) and small deletions or insertions close to codon 600; these mutations generally belong to the *RAS*-like mutated tumor group (2014 TCGA molecular classification scheme) Rearrangement AKAP9::BRAF is an intrachromosomal rearrangement (7q) with fusion of the first eight AKAP9 exons to the C-terminal region of BRAF (exons 9–18). The rearrangement leads to constitutive BRAF activation with *BRAF* p.V600E-like molecular features (2014 TCGA classification)	*BRAF* p.V600E -Papillary carcinoma (40–80%): *BRAF* p.V600E is virtually specific for conventional papillary carcinoma, i.e., classic papillary carcinoma and other subtypes, with the exclusion of tumors belonging to the Encapsulated follicular variant papillary carcinoma family that belongs to the RAS-like mutated group -High-grade non-anaplastic carcinoma of follicular cells: high-grade papillary carcinoma, often belonging to aggressive papillary carcinoma subtypes (e.g., tall cell, hobnail) (> 50%) -Anaplastic carcinoma (10–50%) AKAP9::BRAF Papillary carcinomas that are radiation-induced or that develop in children	*BRAF* p.V600E is widely used as a diagnostic papillary carcinoma marker for preoperative fine needle aspiration molecular typing of cytologically indeterminate thyroid nodules Prognosis: *BRAF* p.V600E has been linked to extrathyroid tumor extension and to an increased risk of PTC recurrence. However, its overall relevance for risk stratification is probably limited when other parameters like stage and histologic typing are taken into account Anaplastic and aggressive radioactive iodide refractory *BRAF* p.V600E mutated thyroid carcinomas respond to a combination of RAF and MEK kinase inhibitors	Ciampi et al. [64]; Cancer Genome Atlas Research Network [50]; Acquaviva et al. [28]; de Biase et al. [65, 66]; Xu et al. [62]; Xu et al. [63]; Subbiah et al. [67]
*CTNNB1*	*CTNNB1* encodes beta-catenin, a cell adhesion molecule and a transcription factor of WNT signaling Mutations may affect all exons, but those that involve the phosphorylation site lead to protein stabilization. Oncogenic activity is due to *CTNNB1* mutations that stabilize the protein by preventing its degradation, followed by the transfer of beta-catenin to the nucleus where it dysregulates transcription. Exon 3 mutations are the most common	Cribriform morular thyroid carcinoma is characterized by crucial mutations in the WNT signaling pathway that are phenotypically equivalent: *APC*, *CTNNB1*, *AXIN1*. In familial adenomatous polyposis patients, the tumor develops due to germline *APC* mutation typically followed by additional *APC* mutations or mutations in *CTNNB1* that cause biallelic gene alteration of WNT signaling. In sporadic cases, biallelic oncogenic alteration of WNT signaling is due to a combination of somatic mutations that most commonly affect *CTNNB1*, associated with *APC*, or *AXIN1* mutations	Beta-catenin accumulation in the cytoplasm and strong nuclear immunoreactivity are the hallmark of oncogenic WNT signaling All patients with cribriform morular thyroid carcinoma should be evaluated for germline APC mutations, since the diagnosis of thyroid carcinoma may predate that of familial adenomatous polyposis in up to ~ 40% of cases	Cameselle-Teijeiro et al. [68]
*DICER1*	*DICER1* encodes an RNA endonuclease (Dicer) involved in the post-transcriptional gene expression of over 30% of protein-coding genes by modulating microRNA (miRNA) and small interfering RNA (siRNA) maturation. *DICER1* mutations are mutually exclusive with other RAS-like and BRAF p.V600E-like mutations -Acquired somatic mutations in nucleotides that encode the catalytic RNase IIIb domain of *DICER1* (Exons24-25) in allele 1: mutations preferentially clustered at 5 specific “hotspot” codons within the RNase IIIb domain that have a key role for miRNA processing -Loss-of-function variant in allele 2 (either germline or somatic): mutations span the coding region of the gene	Thyroid nodules/tumors: ~ 1–2% of cases (overall), but common in children/young adults; abundant colloid, “intermediate” type nuclei with features in between those of follicular adenoma/carcinoma and papillary carcinoma (nuclei are round with coarsely granular chromatin); follicular patterned but may have papillary architecture. *DICER1* mutations are not found in conventional papillary carcinoma High-grade non-anaplastic carcinoma of follicular cells in children, typically poorly differentiated thyroid carcinoma (up to ~ 80%) Thyroblastoma: pathogenic somatic *DICER1* mutations in all tested cases	*DICER1* syndrome (OMIM 601200) is an autosomal dominant tumor predisposition syndrome caused by germline *DICER1* mutation characterized by a wide spectrum of tumors (including pleuropulmonary blastoma, cystic nephroma, Sertoli-Leydig cell tumor); thyroid nodules/tumors are the most prevalent clinical manifestation of *DICER1* syndrome and usually occur by age 40 *DICER1* syndrome should be suspected in children/young adults with thyroid nodules/tumors thyroid tumors that are follicular patterned or with papillary architecture	Rooper et al. [30]; Chernock et al. [69]; Agaimy et al. [70]; Chong et al. [27]; González et al. [29]
*EIF1AX*	*EIF1AX* encodes a protein that mediates the transfer of Met-tRNAf to 40S ribosomal subunits to form the 40S preinitiation complex for protein translation. Mutations of residues on the RNA-binding surface of *EIF1AX* are reported to cause defects in proper 43S and 48S preinitiation complex formation. *EIF1AX* mutations belong to the RAS-like mutated tumor group (2014 TCGA molecular classification scheme)	Follicular pattered thyroid nodules/tumors (follicular adenoma and carcinoma, encapsulated follicular variant papillary carcinoma and NIFTP, hyperplastic nodules of follicular nodular disease/multinodular hyperplasia/thyroid goiter (< 10%)) High-grade non-anaplastic carcinoma of follicular cells, typically poorly differentiated thyroid carcinoma (5–15%) Anaplastic carcinoma (5–15%)	*EIF1AX* mutations coexist with *RAS* mutations in aggressive thyroid carcinoma types (high-grade anaplastic and non-anaplastic carcinoma)	Cancer Genome Atlas Research Network [50]; Xu et al. [62]; Xu et al. [54, 63]
*GLIS1 and GLIS3*	*GLIS1* and *GLIS3* (Glioma-associated oncogene, GLI-similar) are genes belonging to a subfamily of Krüppel-like zinc finger transcription factors that regulate the transcription of a variety of genes in physiological and pathological conditions. *GLIS3* is involved with the activation of the Sonic Hedgehog pathway and regulates thyroid hormone synthesis. Both genes are fused with *PAX8*, in both instances fusion is in-frame and downstream of exon 2 of *PAX8*: the breakpoint involves exon 3 of *GLIS3* and exon 2 of *GLIS1*	Hyalinizing trabecular tumor (~ 100%). PAX8::GLIS3 represents > 90% of all *GLIS* rearrangements	*GLIS* rearrangements are highly specific and are not identified in other tumor types *GLIS* rearrangement detected in preoperative fine needle aspiration is virtually diagnostic for Hyalinizing trabecular tumor *GLIS* fusions induce GLIS overexpression that can be identified by immunohistochemistry	Nikiforova [32]
*EZH1*	*EZH1* (enhancer of zeste homologue 1) encodes a catalytic subunit of the polycomb complex mediating methylation of histone H3 and functioning in the maintenance of embryonic stem cell pluripotency and plasticity. A recurrent hotspot mutation (c.1712A > G; p.G571R) occurs with high frequency in hyperfunctional thyroid nodules/adenomas of adult patients. *EZH1* mutation is almost always found in combination with *TSHR* or *GNAS* mutations or with other presumed alterations in cAMP pathway genes	Hyperfunctional thyroid nodules/adenomas often featuring papillary architecture, so-called “Plummer adenoma”(~ 30%), typically in association with *GNAS* or *TSHR* mutation	*EZH1* mutation does not seem to occur in thyroid carcinomas	Calebiro et al. [31]
*GNAS*	Activated by G-protein-coupled receptors, such as the TSH receptor, GNAS (stimulatory G-protein α-subunit, Gs-alpha) proteins activate membrane-bound adenylate cyclase to produce cAMP. Mutational hotspots in codons 201 and 227, corresponding to the GTP-binding domain, cause constitutive activation of the protein. May be associated with *EZH1* mutations	Hyperfunctional thyroid nodules/adenomas often featuring papillary architecture, so-called “Plummer adenoma” (~ 5%)	*GNAS* activating mutation germline mosaicism occurs in patients with McCune Albright syndrome	Lumbroso et al. [34]; Krohn et al. [33]; Calebiro et al. [31]
*NTRK1 and NTRK3*	Both are transmembrane receptor tyrosine kinases. NTRK1 binds NGF, NTRK3 (a.binds NT3). Both are expressed in the nervous system, where they regulate neuronal differentiation and survival. They are not normally expressed in follicular cells. The tyrosine kinase domain of *NTRK1* is fused to the 5′-terminal regions of heterologous genes (*TMP3*, *TPR*, and *TFG*), and that of *NTRK3* (exon 14) is typically fused to the heterologous gene *ETV6* (exon 4). Rearrangements cause NTRK1 and NTRK3 tyrosine kinase expression in follicular cells in a constitutively active form	Conventional papillary carcinoma (up to ~ 5%, overall). Common in papillary carcinoma of children and young adults (*NTRK3* fusion, ~ 15%; *NTRK1* fusion, ~ 5%). *ETV6::NTRK3* common in radiation-associated papillary carcinoma. Tyrosine kinase fusion papillary carcinoma, in particular *NTRK* and *RET* fusion carcinomas, are characterized by multilobulated/multinodular growth, intratumoral fibrosis, complex papillary architecture with glomerulid papillae and/or solid-trabecular growth, and prominent invasion of vascular spaces, particularly lymphatics Gene fusions (mostly *RET, NTRK, ALK*) are identified in a minority of aggressive thyroid carcinomas: 5–25% of high-grade non-anaplastic carcinoma — typically high-grade papillary carcinoma, up to ~ 5% of anaplastic carcinomas Secretory carcinoma of the thyroid characterized by *ETV6::NTRK3* (~ 100%)	Papillary carcinoma is one of the few epithelial cancers that develop in children, where it is strongly linked to chromosomal rearrangements (mostly *RET*, *NTRK3*, and *NTRK1* fusion), even in the absence of a known history of radiation exposure. In spite of aggressive clinical presentation, papillary carcinoma in children is typically associated with excellent prognosis Therapeutic implications: patients with *NTRK1* and *NRTK3*-rearranged tumors respond to targeted therapy with NTRK inhibitors	Prasad et al. [71]; Dogan et al. [36]; Pekova et al. [37]; Chu et al. [35]
*PIK3CA*	*PIK3CA* encodes the p110α-alpha catalytic subunit of class IA PI3K; PI3Ks are signal transducer enzymes that phosphorylate the position 3 hydroxyl group of the inositol ring of PIP3. PIP3 then activates AKT in the PI3K/PTEN/AKT pathway. Activating *PIK3CA* mutations p.E542K, p.E545K, and p.H1047R are clustered in the helical domain (exon 9) or in the kinase domain (exon 20)	Follicular carcinoma (< 10%) High-grade non-anaplastic carcinoma of follicular cells (< 15%) Anaplastic carcinoma (5–25%)	“Late” molecular event associated with tumor progression	Xu et al. [62]; Xu et al. [54, 63]
*PPARG*	PPARG is a nuclear receptor protein. The complex formed by PPARG receptor, RXRs, and other cofactors activates the transcription of target genes. PAX8–PPARG is involved in tumorigenesis as a consequence of rearrangements with genes that drive the expression of the chimeric PPARG fusion oncoprotein. Other genes may be fused with *PPARG*, such as *CREB3L2*. *PPARG* fusion genes belong to the RAS-like mutated tumor group (2014 TCGA molecular classification scheme)	Follicular adenoma (5–20%) Follicular carcinoma (10–50%) Encapsulated follicular variant papillary carcinoma, invasive and non-invasive (i.e., NIFTP) (0–30%)	*PPARG* fusions induce PPARG overexpression that can be identified by immunohistochemistry Typically found in follicular pattered thyroid tumors (follicular adenoma and carcinoma and encapsulated follicular variant papillary carcinoma family). Uncommon in aggressive thyroid tumors (high-grade anaplastic and non-anaplastic carcinoma)	Kroll et al. [72]
*PTEN*	PTEN (phosphatase and tensin homolog) is a tumor suppressor phosphatase that inhibits AKT activity converting PIP3 to PIP2 and thus negatively regulates the PI3K/PTEN/AKT pathway. The gene is inactivated by mutations, deletions, and epigenetic modifications. Decreased mRNA or protein levels due to epigenetic modifications are more frequent than gene mutations	Follicular carcinoma (< 10%) High-grade non-anaplastic carcinoma of follicular cells is more common in poorly differentiated carcinoma (5–20%) than in high-grade papillary carcinoma (< 5%) Anaplastic carcinoma (10–15%)	“Late” molecular event associated with tumor progression PTEN inactivation can be identified by immunohistochemistry Cowden syndrome (OMIM 158350) is an inherited, autosomal dominant hamartomatous disorder characterized by an increased risk for the development of breast, thyroid, and endometrial carcinoma. It is caused by germline inactivation of *PTEN*. Patients with Cowden syndrome develop hyperplastic/adenomatous follicular-patterned thyroid nodules/tumors including follicular adenoma and carcinoma Cowden syndrome should be suspected in children/young adults with thyroid nodules/tumors that are follicular patterned	Liaw et al. [73]; Harach et al. [74]; Xu et al. [62]; Xu et al. [54, 63]
*RAS*	*HRAS*, *KRAS*, and *NRAS* are G-proteins that have a critical role in the intracellular transduction of signals from the cell membrane. They activate the MAPK pathway, upstream of RAF proteins including (BRAF). Activating *RAS* mutations occur at hotspots in exon 2 (codons 12 and 13) and exon 3 (codon 61). Mutations in the GTPase domain (codon 61) or in the GTP-binding domain (codons 12 and 13) lock the protein in the active GTP-bound form. Mutations occur in any of the *RAS* genes (*NRAS* > *HRAS* > *KRAS* in nodules/tumors of follicular cells, *HRAS* > *KRAS* > *NRAS* in medullary carcinoma). In tumors of follicular cells, mutations most frequently affect NRAS codon 61, and *RAS* mutations are the prototype of the *RAS*-like mutated tumor group (2014 TCGA molecular classification scheme)	Follicular adenoma (20–40%) Follicular carcinoma (30–50%) Encapsulated follicular variant papillary carcinoma, invasive and non-invasive (i.e., NIFTP) (25–45%) High-grade non-anaplastic carcinoma of follicular cells (20–50%) is more common in poorly differentiated carcinoma than in high-grade papillary carcinoma Anaplastic carcinoma (10–50%) Medullary carcinoma (10–20% of sporadic cases, rare in cases with germline RET mutation)	*RAS* mutations can occur in any follicular pattered thyroid nodule/tumor. The presence of *RAS* mutation is generally considered molecular evidence of neoplasm, although mutations are found in a minor subset of histologically hyperplastic nodules of follicular nodular disease/multinodular hyperplasia/thyroid goiter *RAS* mutations are widely used as diagnostic markers for preoperative fine needle aspiration molecular typing of cytologically indeterminate thyroid nodules. The identification of *RAS* mutations indicates that the nodule is likely neoplastic, but by no means not necessarily malignant — as a matter of fact, after thyroid lobectomy, most *RAS*-mutated nodules are diagnosed as benign: hyperplastic nodule, follicular adenoma, or NIFTP	Ciampi [75]; Cancer Genome Atlas Research Network [50]; Acquaviva et al. [28]; de Biase et al. [65, 66]; Xu et al. [62]; Xu et al. [63]
*RET*	*RET* is a transmembrane receptor tyrosine kinase that binds ligands of the glial cell line-derived neurotrophic factor ligand family (GDNF, neurturin, artemin, and persephin) in conjunction with coreceptors, to form a cell surface complex (RET dimer–ligand-coreceptor) that activates downstream signaling, including the MAPK and PI3K/PTEN/AKT pathways. It is expressed in parafollicular calcitonin-producing thyroid C cells. It is not normally expressed (or is expressed at very low levels) in follicular cells Activating mutations Somatic and germline mutations in exons 8–11 and 13–16 are found in medullary carcinoma, familial medullary carcinoma, and MEN2A and MEN2B syndromes. Mutations involve the extracellular cysteine-rich domain (e.g., p.C634R), the intracellular domain (e.g., p.S891A), or the intracellular kinase domain (e.g., p.M918T), causing constitutive activation of RET as protein dimers and/or monomers Rearrangement *RET* fusion genes are due to rearrangements that involve the *RET* locus at 10q11.2. Rearrangements can be intrachromosomal, such as those that produce the two most common forms of RET fusion *CCDC6::RET* (RET/PTC1) and *NCOA4::RET* (RET/PTC3), or interchromosomal, such as *PRKAR1A::RET* (RET/PTC2) and other less common forms. *RET* rearrangement causes RET tyrosine kinase expression in follicular cells in a constitutively active form. *RET* fusion carcinomas belong to the *BRAF* p.V600E-like mutated tumor group (2014 TCGA molecular classification scheme)	*RET* mutation *RET* mutations are present in 40–50% of sporadic medullary carcinoma (RET p.M918T in ~ 70–80% of the *RET*-mutated cases) and often occur in advanced tumors with poor prognosis. Somatic *RET* mutations are rare in medullary carcinoma of patients with germline *RET* mutation *RET* rearrangement -Conventional papillary carcinoma (5–25%, overall). Common in papillary carcinoma of children and young adults and in radiation associated papillary carcinoma. Tyrosine kinase fusion papillary carcinoma, in particular, *NTRK* and *RET* fusion carcinomas are characterized by multilobulated/multinodular growth, intratumoral fibrosis, complex papillary architecture with glomeruloid papillae and/or solid-trabecular growth, and prominent invasion of vascular spaces, particularly lymphatics -Gene fusions (mostly *RET*, *NTRK*, *ALK*) are identified in a minority of aggressive thyroid carcinomas: 5–25% of high-grade non-anaplastic carcinoma — typically high-grade papillary carcinoma, up to ~ 5% of anaplastic carcinomas	All patients with medullary carcinoma must undergo germline screening for *RET* mutation to rule out familial medullary carcinoma, MEN2A (OMIM 171400 for both conditions) or Men2B (OMIM 162300) There is a strong genotype–phenotype correlation between the type of *RET* mutation and clinical manifestations, and depending on the type of *RET* mutation identified, prophylactic thyroidectomy must be considered for the relatives of the proband with *RET* germline mutation Papillary carcinoma is one of the few epithelial cancers that develop in children, where it is strongly linked to chromosomal rearrangements (mostly *RET*, *NTRK3*, and *NTRK1* fusion), even in the absence of a known history of radiation exposure. In spite of aggressive clinical presentation, papillary carcinoma in children is typically associated with excellent prognosis Therapeutic implications: patients with *RET*-mutated medullary carcinoma or with *RET*-rearranged carcinomas of follicular cells respond to targeted therapy with RET inhibitors	Pekova et al. [37]; Wells et al. [13]
*TERT*	*TERT* is the enzyme core protein of the telomerase complex responsible for telomere elongation, which prevents cell senescence and maintains chromosomal integrity and genome stability. *TERT* is expressed in germ cells and somatic stem cells. It is not normally expressed (or is expressed at very low levels) in most somatic cells. Hotspot mutations in the *TERT* promoter are at positions –124 (C228T) and –146 (C250T) upstream of the *TERT* translation start site; *TERT* C228T is much more common than *TERT* C250T. Mutations create a novel binding site for transcription factors of the ETS family, which increase TERT transcription and telomerase expression	Follicular carcinoma (10–35%) Papillary carcinoma, conventional (5–15%) Encapsulated follicular variant papillary carcinoma (5–15%) High-grade non-anaplastic carcinoma of follicular cells (20–50%), equally common in poorly differentiated carcinoma and high-grade papillary carcinoma Anaplastic carcinoma (30–75%)	“Late” molecular event associated with tumor progression *TERT* promoter mutations are clonal and highly prevalent in aggressive carcinomas, while they are uncommon and often subclonal in conventional papillary and follicular carcinoma. Tumors where *TERT* promoter mutation coexists with *RAS* and *BRAF* p.V600E have worse prognosis Powerful indicator of poor prognosis in carcinomas of follicular cells, independent of histologic typing/subtyping and other relevant clinicopathologic parameters	Landa et al. [56]; Xu [62]; Xu et al. [63]; Park et al. [76]
*TP53*	TP53 is a transcription factor involved in the control of the cell cycle, apoptosis, and maintenance of genetic stability. Its levels are controlled by proteasome-dependent degradation. It is the tumor suppressor gene most commonly inactivated in human cancers. Most mutations are missense and affect the DNA binding domain of the protein. Small nucleotide changes in *TP53* are typically located in residues 92–292; exons 5–9 are those usually screened for mutation analysis. The inactive protein is not able to arrest the proliferation of cells with DNA damage	High grade non anaplastic carcinoma of follicular cells (10–35%), more common in poorly differentiated carcinoma compared to high grade papillary carcinoma Anaplastic carcinoma (40–80%) *TP53* mutations have been reported in a minority of oncocytic carcinomas in the absence of high-grade/poorly differentiated features	“Late” molecular event associated with tumor progression, strongly associated with anaplastic carcinoma	Evangelisti et al. [77]; Landa et al. [56]; Xu et al. [62]; Xu et al. [63]
*TSHR*	TSHR is a member of the G-protein-coupled receptor family. It has a complex structure, with a serpentine transmembrane domain with seven loops. Expressed in thyroid follicular cells Mutated *TSHR* and *GNAS* (stimulatory G-protein α-subunit) proteins are constitutively active, resulting in increased intracellular cAMP levels and continuous stimulation of thyroid hormone synthesis and secretion. May be associated with *EZH1* mutations	Hyperfunctional thyroid nodules/adenomas often featuring papillary architecture, so-called “Plummer adenoma” (50–80%)	Inactivating *TSHR* gene mutations are a cause of familial congenital hypothyroidism with thyroid dysgenesis/hypoplasia	Parma et al. [78]; Krohn et al. [33]; Calebiro et al. [31]

i. Genetic alterations include “Early/Driver” molecular changes and “Late/Progression associated” events [28, 50, 62, 63, 79]. These are illustrated in Figs. 2 and 3. Examples of tumors with “Early/Driver” alterations are shown in Fig. 4.

**Fig. 2:**
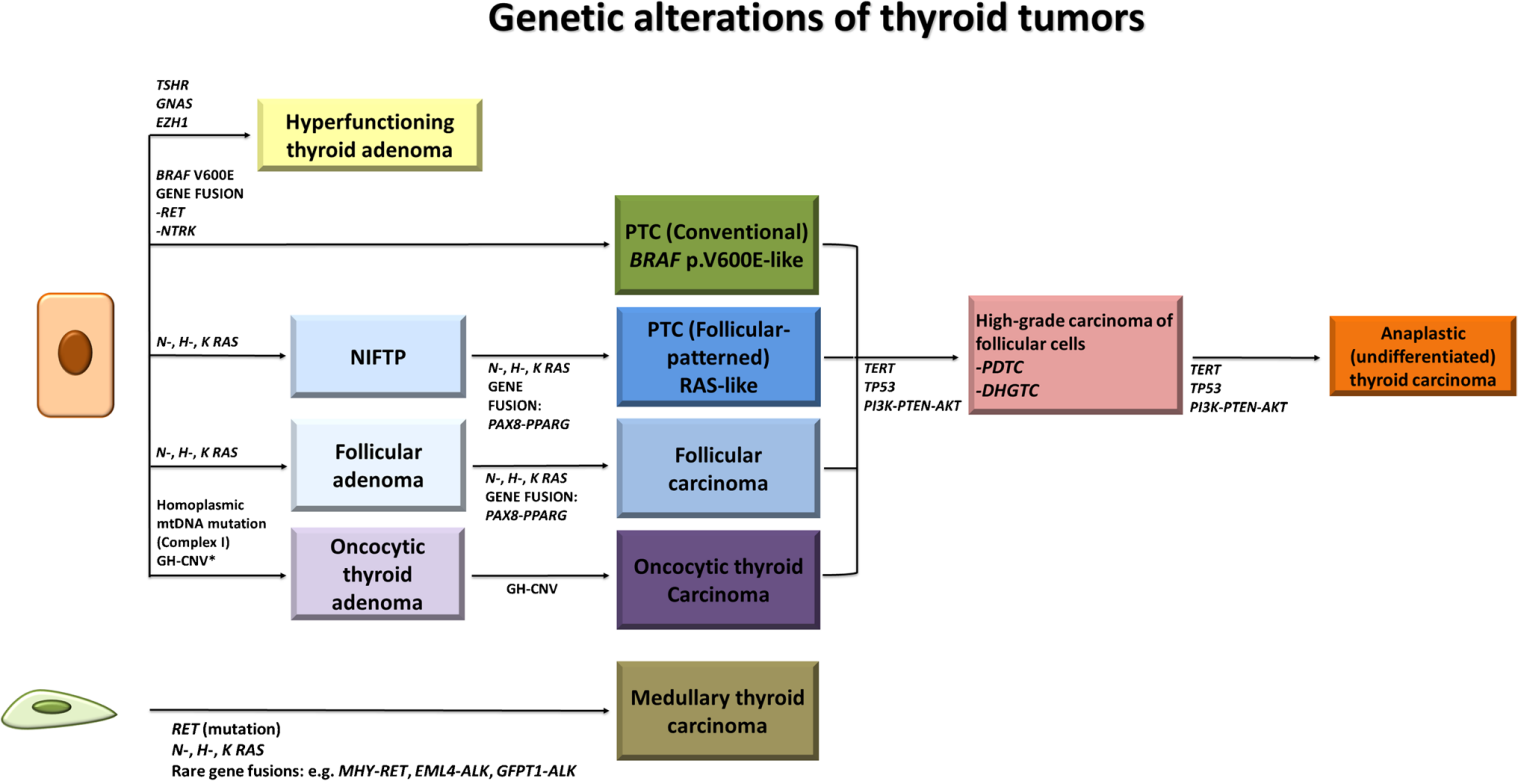
Genetic alterations of thyroid tumors. Genetic alterations include “Early/Driver” molecular changes (*BRAF* p.V600E-like for conventional papillary carcinoma, *RAS*-like for follicular patterned tumors, and coexistence of mtDNA mutations with severe DNA copy-number alterations for oncocytic tumors) as well as “Late/Progression-associated” molecular changes such as *TERT* promoter mutation, *TP53* mutation, alterations of the PI3K/PTEN/AKT pathway in high-grade non-anaplastic carcinoma of follicular cells, and anaplastic thyroid carcinoma. PDTC, poorly differentiated thyroid carcinoma; DHGTC, differentiated high-grade non-anaplastic thyroid carcinoma; GH-CNV, genome haploidization-type DNA copy number variation leading to copy number neutral uniparental disomy. Modified from: Acquaviva G. et al. [28]

**Fig. 3:**
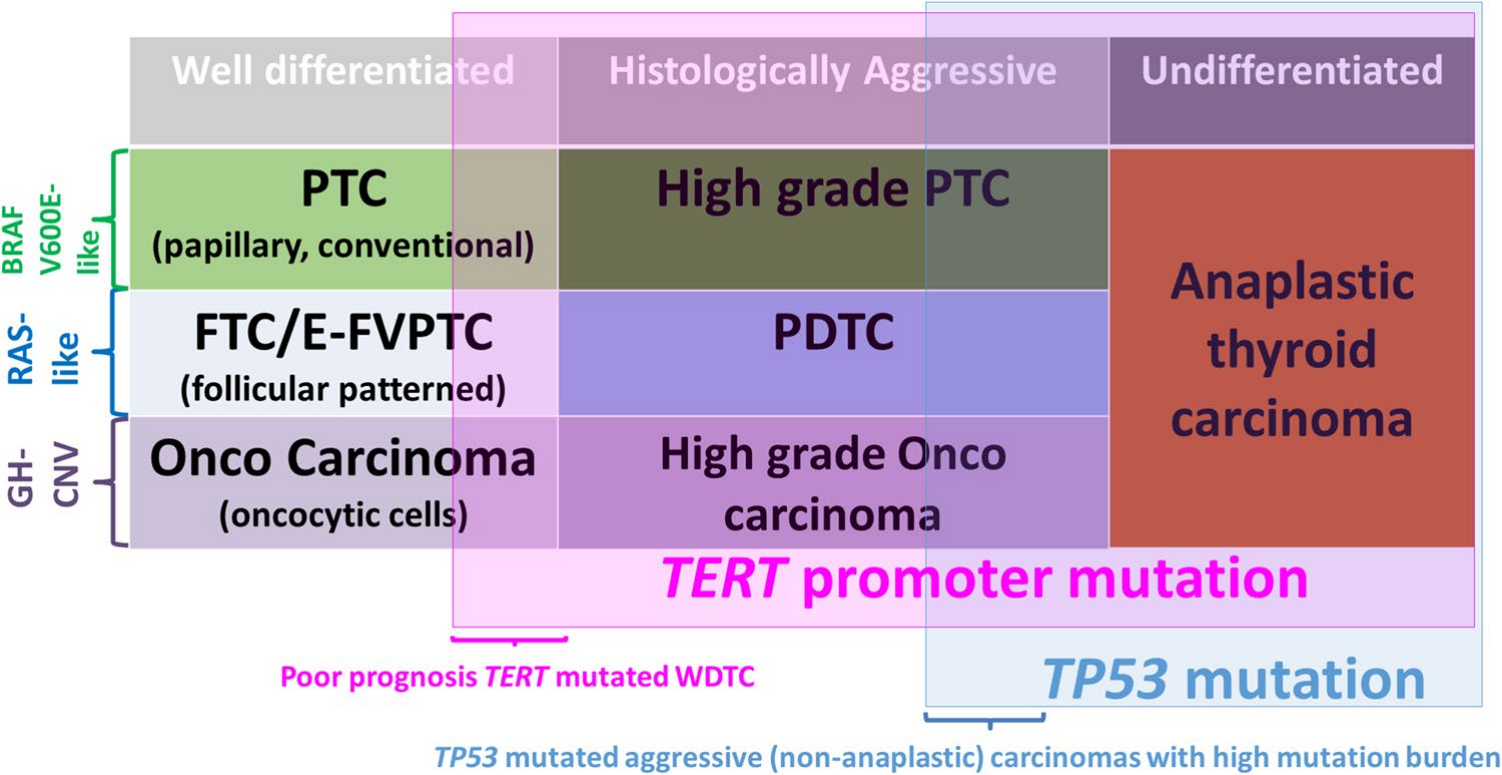
Driver molecular alterations, tumor type, and progression in thyroid tumors of follicular cells. PTC, papillary thyroid carcinoma; E-FVPTC, encapsulated follicular variant papillary thyroid carcinoma; PDTC, poorly differentiated thyroid carcinoma; GH-CNV, genome haploidization-type DNA copy number variation leading to uniparental disomy/aneuploidy. Modified from: Volante et al. [79]

**Fig. 4:**
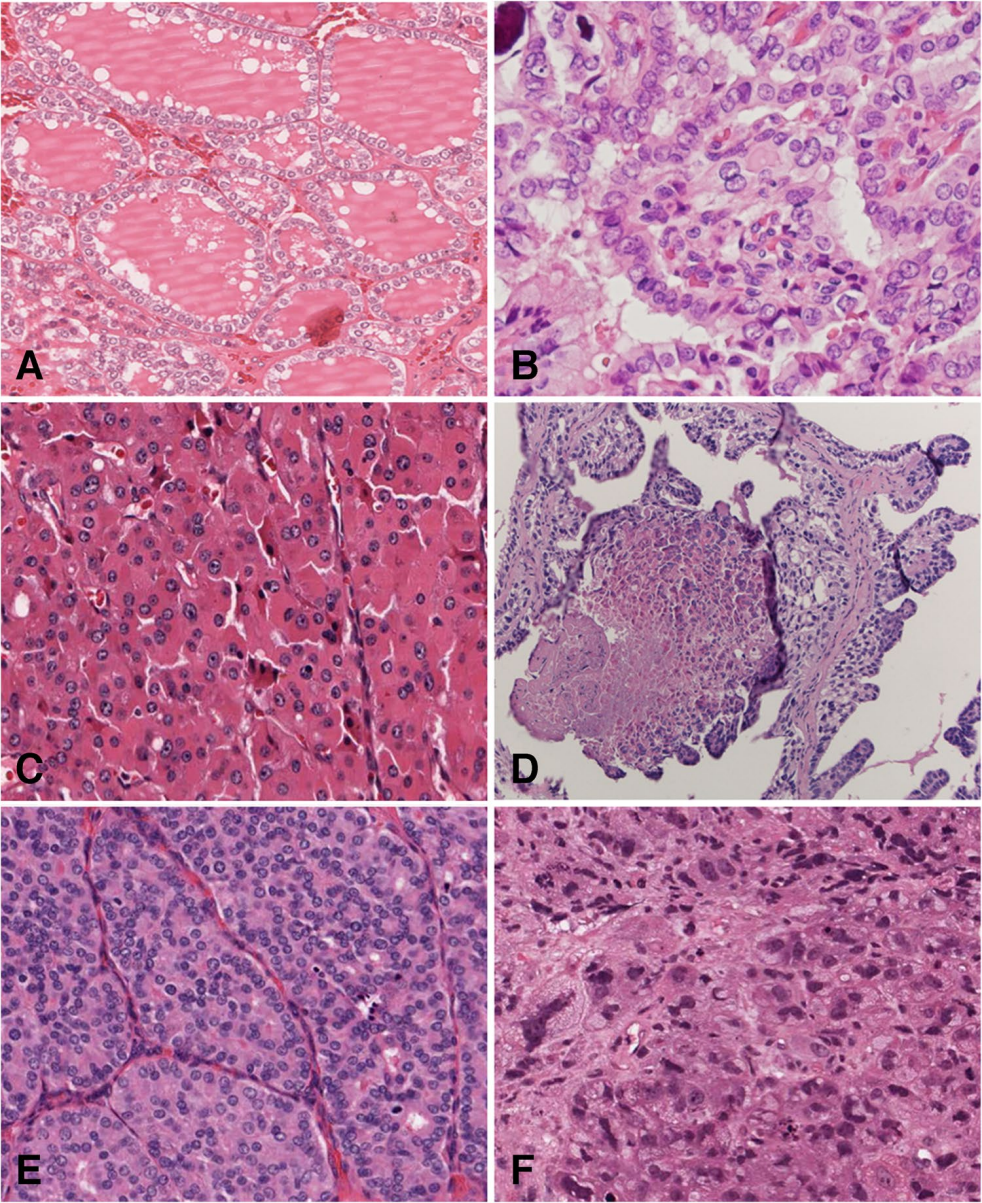
Thyroid tumors. NIFTP (**A**, hematoxylin and eosin): follicular patterned tumors such as NIFTP have *RAS*-like molecular signature. Papillary carcinoma (**B**, hematoxylin and eosin): conventional papillary carcinoma has *BRAF* p.V600E-like molecular signature, this case featuring glomeruloid papillae harbors a *TPR::NTRK1* rearrangement. Oncocytic carcinoma (**C**, hematoxylin and eosin): oncocytic tumors have both mtDNA mutations and severe DNA copy number alterations. High-grade non-anaplastic papillary carcinoma with tumor necrosis (**D**, hematoxylin and eosin), poorly differentiated thyroid carcinoma (**E**, hematoxylin and eosin), and anaplastic thyroid carcinoma (**F**, hematoxylin and eosin): these aggressive high-grade tumors harbor early/driver molecular alterations and additional mutations associated with tumor progression affecting *TERT* promoter and PI3K/PTEN/AKT pathway genes; inactivating *TP53* mutations are typically associated with anaplastic carcinoma
ii. “Early/Driver” alterations are mutually exclusive [28, 50, 62, 63, 79]. They are commonly used for molecular analysis of preoperative fine needle aspiration specimens. The features as well as the pros and cons of the starting material for this type of analysis are summarized in Table 6.

**Table 6:** Starting material for preoperative molecular analysis of thyroid nodules

Starting material	Pros	Cons
Liquid-based cytology	• Good quality of nucleic acid • The Cytological Smears used for morphological diagnosis can be archived	• No evaluation of lesional cells in the analyzed specimen • Usually small amount of starting mater • Not feasible for in situ technique
Cytological smears	• Good quality of nucleic acid • Evaluation of lesional cells in the analyzed specimen • Commonly available for thyroid lesions	• Cover-slide has to be removed • No residual material for archives • Small amount of starting material
Cell block	• Good quality of nucleic acid • Storable in anatomic pathology archives • In situ techniques can be performed	• Small amount of starting material
Surgical FFPE specimen	• Huge amount of material • Representative of the lesions • Storable in anatomic pathology archives • In situ techniques can be performed	• Degradation of nucleic acids due to formalin fixation
Liquid biopsy	• Minimally invasive method • Serially repeatable during follow-up	• Need of very high-sensitive technique • Not feasible for in situ techniques • Technical variability in the pre-analytical and analytical steps
iii. “Late/Progression associated” alterations are found in combination with “Early/Driver” changes, consistent with a general model of multi-step progression from well-differentiated to undifferentiated carcinoma. In cases where poorly or undifferentiated areas are associated with a well-differentiated component, “Early/Driver” alterations are identified in both areas, while “Late/Progression associated” changes are restricted to the less differentiated portions of the tumor [80]. Thus, the number of mutations per tumor increases from well-differentiated to undifferentiated carcinoma. Mutation burden is highest in anaplastic carcinoma, lowest in conventional papillary carcinoma, and intermediate in aggressive/advanced papillary and follicular carcinoma [81]. Examples of tumors with “Late/Progression associated” alterations are shown in Fig. 4.
iv. *RAS* mutations or equivalent molecular alteration (*RAS*-like tumors) are “Early/Driver” events (see paragraph (ii)) for follicular patterned tumors (Figs. 2, 3, and 4 and Table 5). *RAS*-like tumors have a homogeneous molecular profile, low MAPK-signaling (because of *ERK* to *RAF* monomer negative feedback), high differentiation score, and are malignant only if there is an invasion of the tumor capsule or blood vessels [50].
v. *BRAF* p.V600E mutation or equivalent molecular alterations (*BRAF* p.V600E-like tumors) are “Early/Driver” events (see paragraph (ii)) for conventional papillary carcinoma (Figs. 2, 3, and 4 and Table 5). *BRAF* p.V600E-like tumors have a heterogeneous molecular profile, high MAPK-signaling (because of the lack of *ERK* to *RAF* monomer negative feedback), and low differentiation score (based on the level of expression of 16 thyroid metabolism and function genes, e.g., *TG*, *TPO*, *PAX8*).
vi. Coexistence of mtDNA mutations with severe DNA copy-number alterations represents the “Early/Driver” event (see paragraph (ii)) for oncocytic tumors (Figs. 2, 3, and 4 and Table 5). Mutations of mitochondrial DNA (mtDNA) are homoplasmic mtDNA and mostly affect mitochondrial genes encoding Complex I of the respiratory chain [52]. DNA copy-number alterations are dramatic, with widespread loss of heterozygosity and loss of chromosomal DNA, following genome-wide DNA haploidization and copy-number-neutral uniparental disomy [53, 82, 83]. This pathway is unique to oncocytic tumors, which typically do not carry conventional *BRAF-like* or *RAS-like* alterations [82–84]. While mtDNA mutations are responsible for the oncocytic phenotype [52], the loss of chromosomal DNA is linked to tumor development, since genome haploidization-type DNA copy-number changes are more common in cases diagnosed histologically as oncocytic carcinoma as opposed to oncocytic adenomas and are rare in hyperplastic oncocytic nodules. This has potentially important implications for molecular testing of preoperative fine needle aspiration, since conventional *BRAF-like* or *RAS-like* alterations are commonly absent in oncocytic tumors, while Bethesda category III and IV with oncocytic changes have a higher prevalence of DNA copy-number alterations compared with the same categories without cytologically identified oncocytic morphology [85].
vii. “Late/Progression-associated” alterations include mostly somatic mutations of *TP53*, *TERT* promoter, and dysregulation of the PI3K/PTEN/AKT pathway (Figs. 2 and 3 and Table 5). Mutations of *CDKN2A*, of SWI/SNF (switch/sucrose non-fermentable) chromatin remodeling complex genes (*ARID1A*, *ARID1B*, *ARID2*, *ARID5B*, *SMARCB1*, *PBRM1*, *ATRX*), of Histone methyltransferase genes (*KMT2A*, *KMT2C*, *KMT2D*, *SETD2*), and of DNA mismatch repair (*MMR*) genes (*MSH2*, *MSH6*, and *MLH1*) have also been reported [56, 62, 63]. “Late/Progression-associated” changes, in particular *TERT* promoter mutations, can be utilized for risk stratification, also using preoperative fine needle aspiration specimens [57].
viii. *TERT* promoter mutations are more frequent and have higher mutated allelic fraction in poorly differentiated, anaplastic, and aggressive/advanced cancers (including high-grade papillary carcinoma) compared with well-differentiated carcinoma [56, 62, 63].
ix. *TERT* promoter and particularly its co-mutation with *BRAF* p.V600E or *RAS* is a powerful marker of poor outcome. Aggressive/advanced papillary carcinomas, many of which are histologically high-grade, have at least one of three genetic alterations: duplication of chromosome 1q, duplication of chromosome 5 p harboring the *TERT* genomic locus, and *TERT* promoter mutation (THYT1 signature) [57].
x. *TP53* mutation has the highest prevalence in anaplastic carcinoma compared to all forms of advanced/aggressive thyroid carcinoma, including both poorly differentiated and high-grade papillary carcinoma [56, 62, 63, 66].
xi. Rearrangements — such as *RET/PTC*, *NTRK1*, *NTRK3*, and *PAX8-PPRG* not rare in well-differentiated tumors — are uncommon [56, 62, 63].

## Parathyroid tumors: molecular pathology and correlation with clinicopathologic features

The spectrum of parathyroid tumors includes adenoma, atypical tumor (neoplasm of uncertain malignant potential, previously defined as “atypical parathyroid adenoma”), and carcinoma. These tumors can arise in any gland including ectopic ones or areas where embryonic parathyroid remnants may be found [86]. The majority of them cause primary hyperparathyroidism, with adenomas accounting for at least 85% of cases [87, 88].

In general, immunohistochemistry is not necessary for diagnostic purposes. However, it may be useful to screen for hereditary conditions associated with inactivation of genes such as *CDC73* and *MEN1*, causing hyperparathyroidism-jaw tumor (HPTJT) and MEN1 syndrome, respectively [89]. These hereditary conditions usually present with multiglandular involvement and/or multinodular tumors. Parathyroid tumors that arise in the context of hyperparathyroidism-jaw tumor (HPTJT) syndrome feature eosinophilic cytoplasm, perinuclear clearing, nuclear expansion, micro-cystic structures, and sheet-like growth pattern [90].

**Fig. 5:**
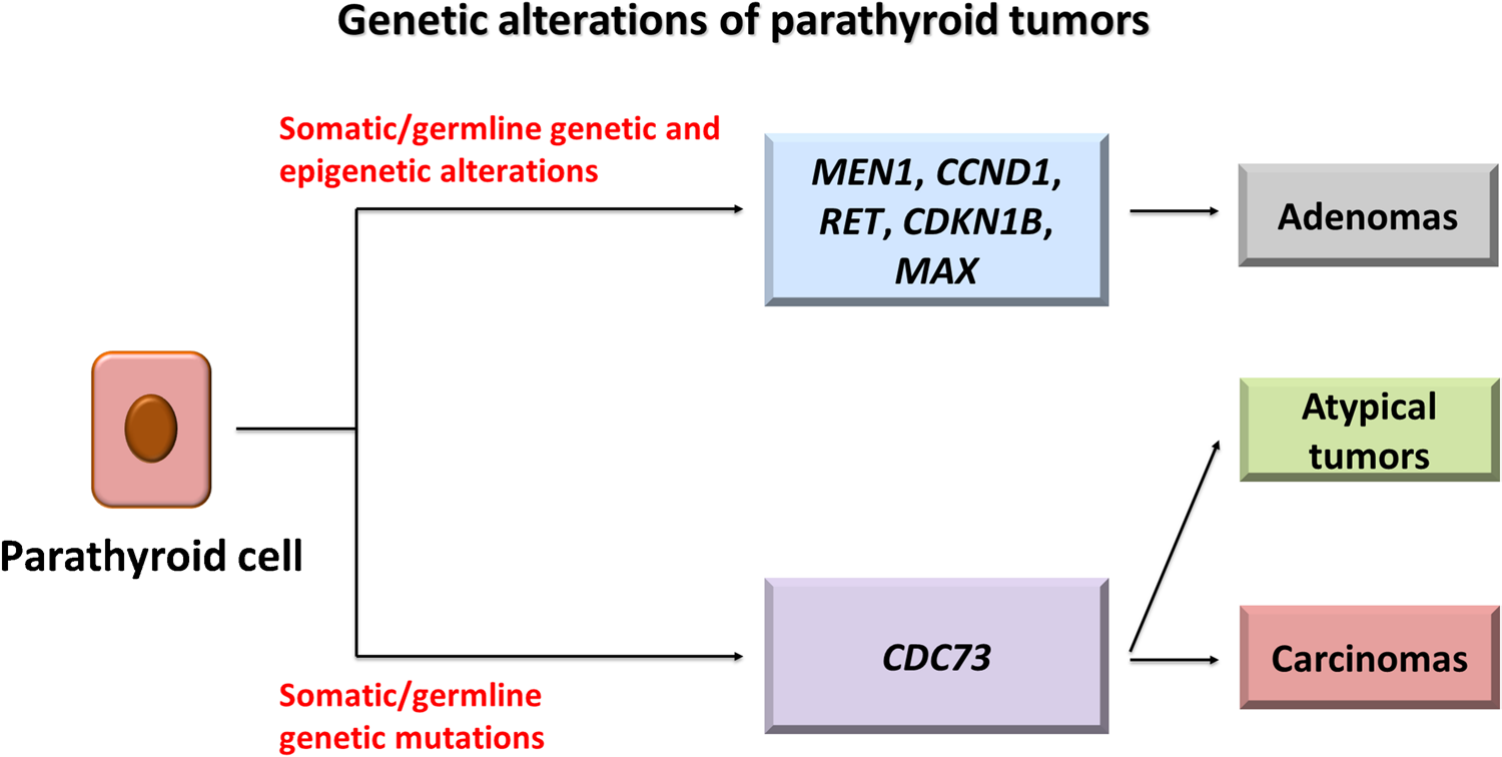
Genetic alterations of parathyroid tumors*. MEN1* loss of function represents the most common alteration in parathyroid adenoma. The most common alteration of parathyroid carcinoma is *CDC73* inactivation, also found in a minority of atypical parathyroid tumors

**Fig. 6:**
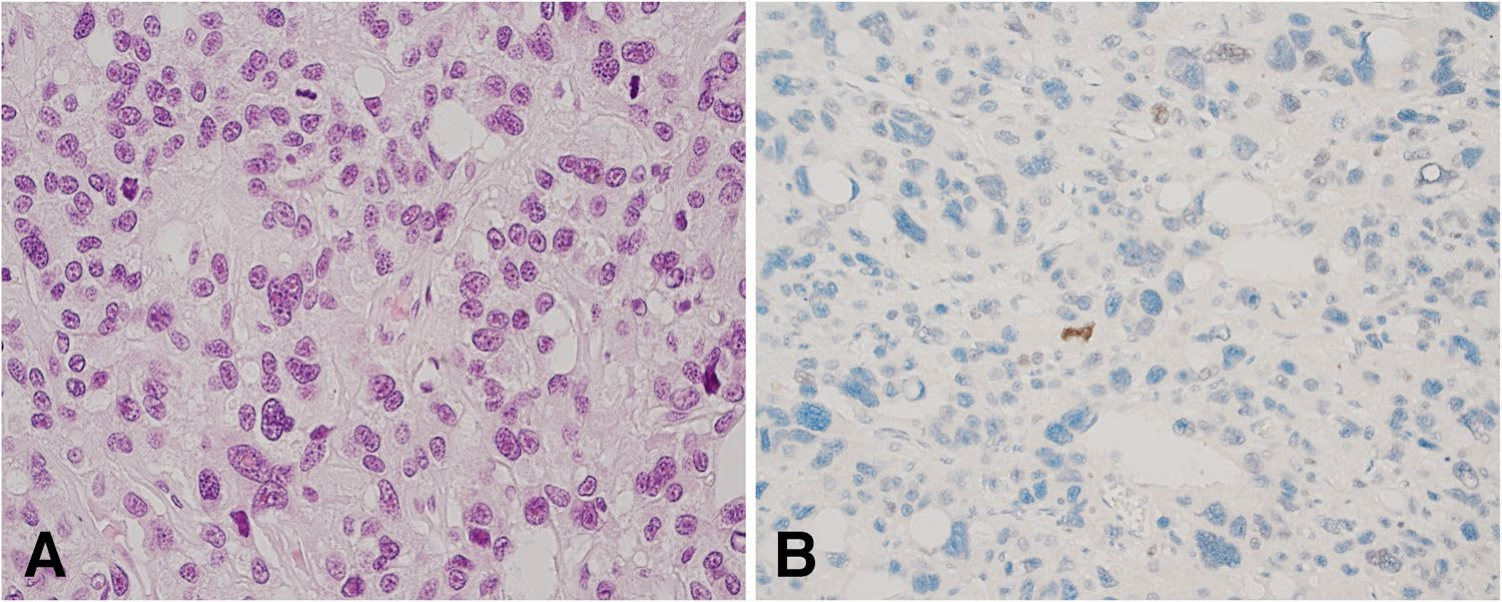
Parathyroid carcinoma. There is mitotic activity, nuclear pleomorphism (**A**, hematoxylin and eosin), and parafibromin expression is lost (**B**, parafibromin immunohistochemistry)

The main genes mutated in parathyroid tumors are summarized in Table 7 and illustrated in Fig. 5. Examples of parathyroid tumors are shown in Fig. 6. The molecular pathogenesis underlying the majority of sporadic parathyroid adenoma remains unknown. Syndromic parathyroid adenomas constitute ~ 10% of cases [91], found in MEN1, MEN2, MEN4, HPTJT, and isolated familial hyperparathyroidism (FIHP). These syndromes have recently been complemented by MEN5, associated with hereditary mutations of *MAX* [92] (Table 1). In these inherited conditions, the parathyroid glands contain multiple clonal adenomas which mimic the clinical appearance of cases traditionally diagnosed as parathyroid hyperplasia [93, 94].

**Table 7:** Somatic genetic alterations of parathyroid tumors and their clinicopathologic relevance

Gene(s)	Molecular pathology	Parathyroid tumor type(s)	Clinicopathologic implications	Reference(s)
*CCND1*	Rearrangement of *CCND1* or gene overexpression induced by epigenetic changes increase Cyclin D1 expression, thus promoting cellular proliferation	Parathyroid adenoma	Therapeutic implications: potential response to CDK4/6 inhibitors	[95]
*CDC73*	Mutations in addition or loss of heterozygosity of the parafibromin encoding gene alter its nuclear-tumor-suppressing function	Parathyroid carcinomas	Genomic instability Unfavorable, prognosis, tumor recurrence	[96–102]
*MEN1*	LOH or inactivating intragenic mutations (nonsense mutations, frameshift deletions or insertions, in-frame deletions or insertions, splice-site mutations, and missense mutations) cause loss of MEN1	Parathyroid adenoma	MEN1 loss is rare in parathyroid carcinoma	[103, 104]

In sporadic adenoma, the most common somatic alteration is inactivation of *MEN1*. This is caused by loss of heterozygosity (LOH) due to large deletions or genetic recombination at 11q13 (where *MEN1* is located) found in ~ 35% of all parathyroid adenoma and/or *MEN1* inactivating mutations found in up to ~ 20% of cases [105]. In addition to LOH and somatic mutations, other mechanisms can lead to the inactivation of *MEN1*, including epigenetic silencing. Interestingly, biallelic *MEN1* inactivation occurs in approximately 50% of cases in which LOH at 11q13 is detected, raising the hypothesis that other genes on 11q may also play a role in tumor development.

Cyclin D1 (also located at 11q13) is overexpressed in 10–40% of parathyroid adenomas due to aberrant promoter methylation of different cyclin-dependent kinase inhibitors (CDKIs), while rearrangement of the Cyclin D1 gene (*CCND1*) occurs in up to ~ 10% of parathyroid adenomas [105].

Other somatic mutations involving *CDKN1B* (encoding p27), *EZH2* (encoding the zinc-finger protein X-linked transcription factor), *ASXL3*, and *MTOR* have been reported in a small minority of parathyroid adenomas [105–107]. Somatic *CDC73* mutations are rare in adenomas. They have been reported only in atypical parathyroid tumors, in some adenomas in the context of HPTJT, and in a small number of cystic adenomas [108]. Interestingly, parathyroid nodules in secondary or tertiary hyperparathyroidism — typically associated with chronic renal failure — harbor different somatic changes compared with those of adenomas in primary hyperparathyroidism [106].

Parathyroid carcinoma is rare and the majority of cases are sporadic. Parathyroid carcinoma develops in 10–15% of patients with HPJT and FIHP (Table 1), while it is uncommon in other inherited conditions [90, 109, 110]. Only a few studies have been conducted on the molecular pathogenesis of sporadic carcinomas. Contrary to parathyroid adenomas, parathyroid cancer rarely exhibits *MEN1* mutations [111]. Inactivating *CDC73* alterations have been reported in 40–80% of sporadic cases [111–113]. *CDC73* alterations include truncating or frameshift mutations, as well as missense mutations leading to the loss of parafibromin immunoreactivity [114]. *CDC73*-mutant parathyroid carcinomas exhibit higher genomic instability with DNA copy number changes, greater mutational burden, and worse patient outcomes compared with wild-type cases [115].

Loss of *TP53* and *RB1* alleles, *CCND1* (encoding Cyclin D1) amplification, and *TERT* promoter mutations have been reported [116, 117]. *PTEN*, *NF1*, *KDR*, and *PIK3CA* mutations may represent potential targets for molecular therapy [118]. Metastatic parathyroid carcinoma has a different expression profile compared with non-metastatic parathyroid carcinoma and parathyroid adenoma [119, 120]. Several epigenetic alterations have been discovered in parathyroid carcinomas, including aberrant methylation of *APC* and of the cell cycle regulators *CDKN2A* and *CDKN2B* [121].

## Adrenal cortical tumors: molecular pathology and correlation with clinicopathologic features

**Fig. 7:**
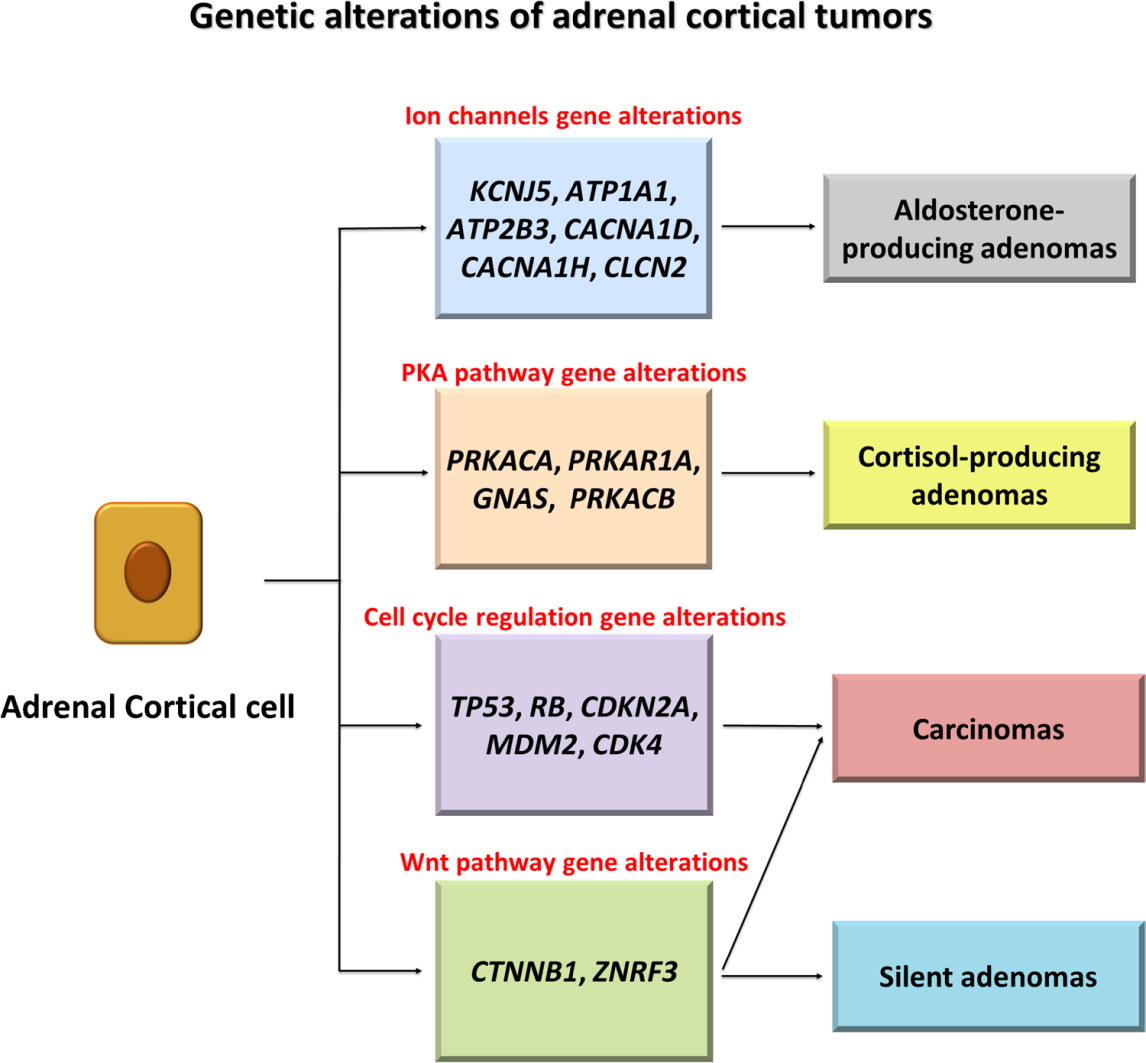
Genetic alterations of adrenal cortical tumors. Adenomas arise as a result of mutations affecting two main groups of genes: the aldosterone-producing adenomas harbor most frequently mutations for *KCNJ5* or the ion channel encoding genes, while cortisol-producing adenomas often develop due to alterations in the PKA pathway, typically *PRKACA*. Genetic alterations of carcinomas mainly involve *TP53* but also genes commonly mutated in non-functioning adenomas

The spectrum of endocrine tumors of the adrenal cortex includes adrenocortical nodular disease, adrenal cortical adenoma, and adrenal cortical carcinoma. Recently, molecular insights have led to modify the terminology related to adrenocortical nodular disease [122] which currently includes several types of clonal benign proliferations: sporadic nodular adrenocortical disease (a common condition), micronodular adrenocortical disease (a rare condition), and bilateral macronodular adrenocortical disease (a rare condition). Micronodular and bilateral macronodular adrenocortical diseases are often associated with germline pathogenic mutations of several genes [123–125]. Our understanding of genomic and hormonal landscapes of adrenal cortical adenoma has also advanced significantly [126], and genotype–phenotype correlations have been proposed for both aldosterone-producing [127, 128] and cortisol-secreting adenomas [129]. Importantly, it is not uncommon for a single alteration to affect different functional pathways, as has been demonstrated for *KCNJ5* mutations [130]. Concerning the pathogenesis of cortical carcinoma, several main pathways of tumorigenesis have been discovered, involving cell cycle regulation, Wnt signaling, chromosome maintenance/chromatin remodeling, and the PKA pathway [131] (Fig. 7).

Furthermore, integrated analysis of these findings with transcriptomic data, epigenetic findings, and copy number changes has led to the identification of three main classes of adrenal cortex carcinoma, with important clinical and prognostic implications [132–134].

### Adrenocortical nodular disease

Nodular adrenocortical disease with bilateral involvement of the adrenal cortex rarely occurs in young patients, but when present, it is frequently associated with germline conditions (Table 1). Germline variants of *PRKAR1A*, *PRKACA*, *PDE11A*, *PDE8B*, and 2p16 *CNC2* locus alterations are frequently reported in micronodular adrenocortical disease with bilateral involvement of adrenal cortex, which typically affects children and young adults [135] (Table 1). Germline *PRKAR1A* mutations (less frequently of *PDE8B* and *PDE11A*) cause Primary Pigmented Nodular Adrenocortical Disease, a distinct subtype of bilateral micronodular adrenocortical disease typically found in association with Carney’s complex [136]. The bilateral macronodular adrenocortical disease is caused by pathogenic *ARMC5* variants (~ 50% of cases). Further alterations may involve the following genes: *MEN1*, *FH* (Hereditary Leiomyomatosis and Renal Cell Cancer), *APC* (Familial Adenomatosis Polyposis), *GNAS* (McCune Albright Syndrome), and the rarely mutated *PDE11A*, *PDE8B*, and 2p16 *CNC2* locus [123, 125, 132, 136, 137]. Variable patterns of Cushing syndrome are typical clinical manifestations of both micro- and macronodular adrenocortical disease.

### Adrenal cortical adenoma

Cortical adenomas are the most common tumors of the adrenal cortex.

Alterations of distinctive pathways (active under normal conditions) involved in the physiologic production of aldosterone and cortisol are typical of the corresponding hormone-producing adenoma. Indeed, functioning adenomas that cause primary aldosteronism harbor specific somatic mutations of several ion channel genes which lead to both cellular proliferation and increased aldosterone production in the cells of the zona glomerulosa [126]. They are mutually exclusive and involve *KCNJ5* (K + channel) [138], *ATP1A1* (Na + /K + channel) [139], *ATP2B3* (Ca2 + channel) [139], *CACNA1D* (Ca2 + channel), *CACNA1H* (Ca2 + channel) [140], and *CLCN2* (Cl- channel) [95, 141]. *KCNJ5* mutated adenomas account for the large majority of the cases (~ 40% of aldosterone-producing adenomas) and tend to mainly affect young female patients [137, 142]. Recurrent phenotypic and clinical characteristics have been identified in ion channel gene mutated adenomas [127, 128]. As shown in Table 8, these include the expression of steroidogenic enzymes, cytomorphology, and lateralization index of the adrenal vein sampling (AVS). Interestingly, ion channel genes such as *KCNJ5*, *CACNA1H*, *CACNA1D*, or *CLCN2* may also be mutated in the germline, causing familial aldosteronism [126]. Somatic mutations of *CTNNB1* — encoding the Wnt-pathway effector beta-catenin — occur in ~ 5% of sporadic aldosterone-producing adenomas and have been correlated to delayed disease onset and female prevalence [96].

**Table 8:** Genetic alterations of adrenal cortical tumors and their clinicopathologic relevance

Gene(s)	Molecular pathology	Adrenal cortical tumor type(s)	Clinicopathologic implications	Reference(s)
*ARMC5*	Frequently germline missense mutations or, rarely, deletion of *ARMC5* inactivate its oncosuppressive function	Bilateral macronodular adrenocortical disease	Adult onset High level of cortisol secretion compared to *ARMC5* wild type disease Adrenal enlargement	[136]
*CTNNB1*	Activating mutations of Beta-catenin lead to its accumulation in the nucleus and downstream activation of the Wnt pathway	Adrenal cortical carcinomas (ACCs) Silent adenomas	ACCs: worst outcomes Silent adenomas: great dimension	[132]
*GNAS*	Somatic mosaicism of *GNAS* or somatic activating mutations promote tumor development	Cortisol-producing adenomas Bilateral macronodular adrenocortical disease (PBMAH)	Adenoma: young patients; pronounced hypercortisolism; small, small tumors PBMAH: McCune-Albright Syndrome; childhood onset; pronounced hypercortisolism and other endocrinopathies	[96–102]
*KCNJ5*	Nucleotide substitutions, insertions, or deletions at the selective site for ${\mathrm{K}}^{+}$ exit cause membrane depolarization, as in the physiological response to angiotensine with aldostereone excess and cell cycle activation	Aldosterone-producing adenomas	High aldosterone levels than in wild type tumors Female patients High dimensions Descending lateralization index response to cosyntropin at AVS	[138]
*PRKACA*	Germline or, more frequently, somatic activating mutations of PKA-C lead to transcription of genes promoting proliferation and cortisol-production	Cortisol-producing adenomas Micronodular adrenocortical disease (MAD)	Adenomas: younger patients; overt Cushing; smaller tumors MAD: early age of onset; multiple small “bead-like” nodules at imaging; paradoxical cortisol response on Liddle’s test; variable degrees of hypercortisolism	[132]
*TP53*	Inactivating mutations cause loss of function of the p53 protein	Adrenal cortical carcinomas (ACCs)	Unfavorable prognosis Advanced stage disease	[100, 132]

Functioning adenomas that produce cortisol feature genetic alterations of the protein kinase A (PKA) pathway active under normal conditions in the production of cortisol. The PKA pathway is physiologically activated by ACTH so that PKA catalytic subunits (PKA-C) can enter the nucleus of zona fasciculata cells enhancing transcription of genes that promote cell proliferation and synthesis of cortisol [126]. PKA pathway genetic alterations of cortisol-producing adenomas affect most frequently the following genes: *PRKACA* (~ 40% of cases), *PRKAR1A*, *GNAS*, or *PRKACB* [97, 98]. As for sexual steroid-producing adenomas, the molecular pathogenesis remains largely unknown. *CTNNB1* mutations are the most frequent molecular alterations of cortical adenomas not associated with hormone production (silent adenomas), particularly in large-sized tumors [129].

### Adrenal cortical carcinoma

**Fig. 8:**
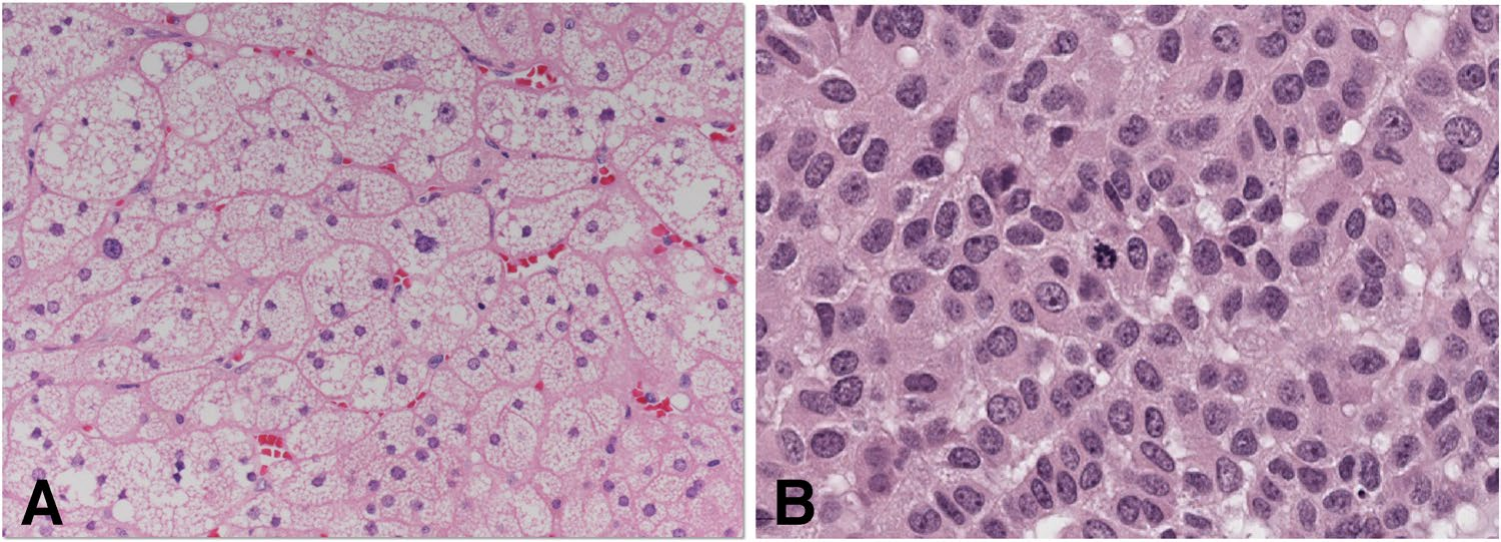
Adrenal cortical tumors. Adrenal cortical adenoma composed by lipid-rich cells resembling the zona fasciculata with low mitotic activity (**A**, hematoxylin and eosin): cortisol-producing adenoma such as the one shown in the picture has PKA pathway alterations. Adrenal cortical carcinoma with nuclear pleomorphism, mitotic activity, and trabecular growth pattern (**B**, hematoxylin and eosin): *TP53* is the gene most commonly mutated

Adrenal cortical carcinoma is rare and mostly associated with somatic genetic changes (Figs. 7 and 8). Common clinical presentations include Cushing or adrenogenital (virilization-feminization) syndromes due to hormone production [99, 122]. Hereditary cases typically affect children, with up to 80% of pediatric cases carrying germline mutations. The most common mutation affects *TP53* (Li-fraumeni syndrome), followed by alterations of the mismatch repair system (Lynch syndrome) [100, 101]. Additional hereditary conditions include Beckwith-Wideman syndrome and MEN1 [100]. Genes frequently altered in benign conditions such as *PRKAR1A*, *MSH2*, *APC*, *MEN1*, and *NF1* are mutated only in small subsets of carcinomas [132]. The most common genetic signatures affect the cell cycle, Wnt signaling, and chromatin remodeling. *TP53* mutations found in ~ 20% of adrenal cortical carcinomas are the most common changes [143]. Recurrent somatic genetic alterations affect other cell cycle regulatory genes such as *RB1*, *CDK2NA* [132], *MDM2*, and *CDK4* [131, 133]. Wnt signaling is dysregulated by *CTNNB1* mutations, *ZNRF3* mutations, and deletions [132, 143]. Importantly, *TP53* or Wnt pathway mutations are typically mutually exclusive but similarly associated with poor prognosis [132, 133, 143]. Dysregulation of chromatin remodeling is caused by the alteration of several genes, such as *ATRX* and *DAXX* [132].

Furthermore, structural alterations (rearrangements and deletions) in tumor DNA at 11p15 are a frequent finding and can cause loss of imprinted *H19* tumor suppressor gene and overexpression of the *IGF2* oncogene [103]. *IGF2* overexpression in adrenal cortical carcinoma can be identified by immunohistochemistry and may be useful in the differential diagnosis with adenoma [104].

Chromosomal alterations are heterogeneous and have been clustered into three groups: those with extensive chromosome loss (~ 50%), those with variable levels of ploidy (~ 40% — the group of chromosomal changes with worse prognosis), and those with limited chromosomal DNA alteration (~ 10%).

Comprehensive molecular classification of adrenal cortical carcinoma is evolving [126, 143]. Over the years, evidence provided by mutation analysis, chromosomal, methylation, and transcriptome profiling has been integrated to define prognostic groups for risk stratification [126, 133, 134, 144]. According to an important study by Assié et al., there are two main types of adrenal cortical carcinoma [132]. The set of “CpG island methylator phenotype-low” (CIMP-low) carcinomas has infrequent alterations in *TP53* or Wnt pathways, mRNA expression pattern predictive of less severe prognosis, chromosome loss, and low rate of disease progression. “CIMP-high” carcinomas typically have alterations of *TP53* or Wnt pathway, mRNA expression pattern predictive of poor prognosis, whole-genome duplication, and high rate of disease progression. The TCGA has built on this experience and has proposed an integrated molecular classification model based on DNA copy number, DNA methylation, mRNA expression, and miRNA expression profiles [132–134]. This classification model has three groups — termed Cluster-1, -2, -3 — which have been defined after Cluster of Cluster (CoC) analysis. Each CoC cluster is highly relevant for patient prognosis, with Cluster 3 being associated with worse outcome [132–134].

## Paraganglionic tumors (tumors of the adrenal medulla and of extra-adrenal paraganglia): molecular pathology and correlation with clinicopathologic features

**Fig. 9:**
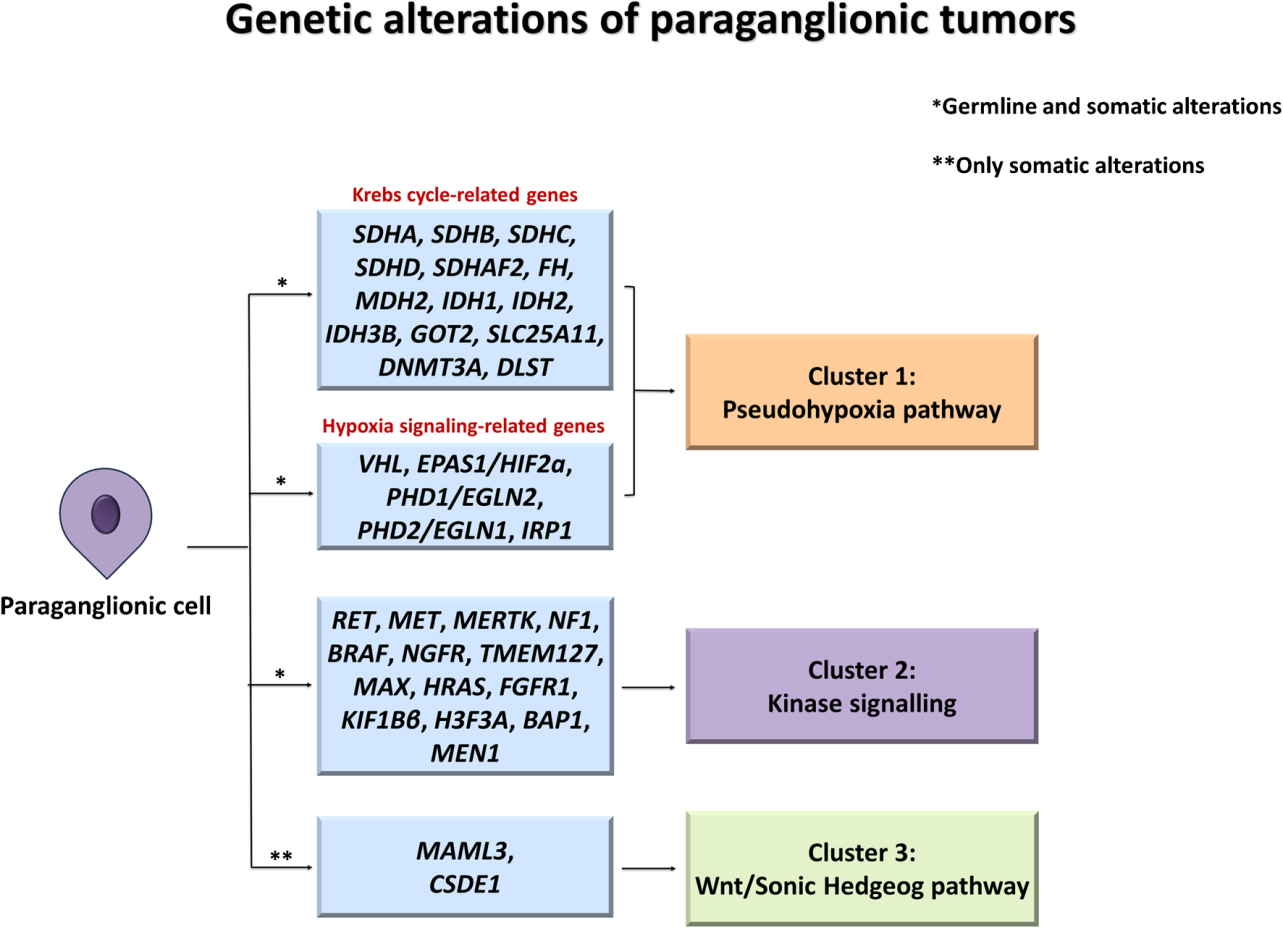
Genetic alterations of paraganglionic tumors. Genetic alterations in PPGL have been grouped into three main groups reflecting three different mechanisms of tumorigenesis: the Cluster 1-pseudohypoxia pathway, characterized by genetic alterations of the HIF1-alpha-activated response to hypoxia pathway and of other similar hypoxia-inducible factor gene pathways; the Cluster 2-Kinase signaling, including the most common alterations identified in paraganglionic tumors such as *NF1* mutations; the more recently described Cluster 3-Wnt/Sonic Hedgehog pathway. *Germline and somatic alterations. **Only somatic alterations

**Fig. 10:**
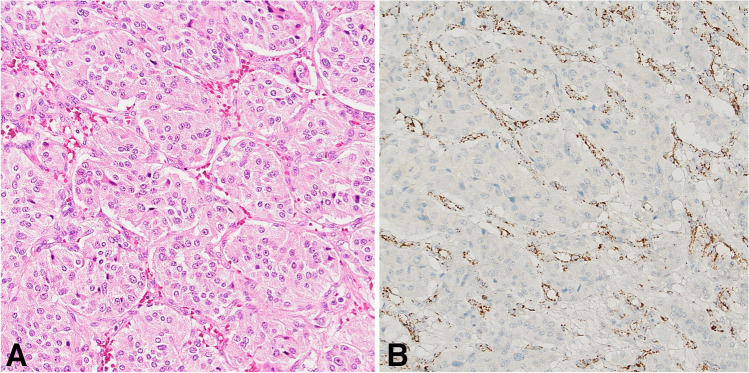
Paraganglionic tumors. Cells resembling normal chromaffin cells with abundant granular cytoplasm are arranged in well-defined nests (**A**, hematoxylin and eosin). Head and neck paraganglioma, such as the one shown in the picture, often has inactivating *SDHD* mutations. If any of the subunits of the SDH complex is altered, the entire complex becomes unstable and immunohistochemical SDHB expression is lost (**B**, SDHB immunohistochemistry)

The main genes mutated in paraganglionic tumors are illustrated in Fig. 9. An example of a paraganglionic tumor is shown in Fig. 10.

Paraganglionic tumors are neuroendocrine neoplasms that develop from neural crest-derived progenitors in the adrenal medulla (Pheochromocytoma) and paraganglia (Paraganglioma), respectively. In the latest WHO classification of endocrine tumors, they are considered malignant, although the overall proportion of cases that metastasize is low, ~ 10% of cases. Paragangliomas are further classified in sympathetic and parasympathetic neoplasms according to cell origin and localization. In particular, sympathetic paraganglioma arises within sympathetic nerve plexuses, fibers, and pre- and paravertebral sympathetic chains — thus abdominal cavity, retroperitoneum, pelvis, and thorax are the most prevalent sites. The majority of parasympathetic paraganglioma develops from parasympathetic glomera, and tumors are typically found in the head and neck region, while pheochromocytoma develops in the adrenal medulla. Paraganglionic tumors share the same embryonic origin and are frequently associated with inherited germline mutations (Table 1). Indeed, paraganglionic tumors have the highest degree of hereditability among human neoplasms, with germline mutations in up to ~ 40–80% of cases (vs. ~ 10% in other tumor types) [145–147]. Most cases of sympathetic paraganglionic tumors are functional due to catecholamine production, causing hypertension in the majority of patients [148], while parasympathetic paraganglioma typically presents as asymptomatic masses. Sympathetic paraganglionic tumors in children account for up to 20% of cases [149, 150] and most are associated with germline mutations [102, 150]. Conversely, parasympathetic paragangliomas rarely affect children [102, 150, 151]. Germline mutations are an important risk factor for the development of all types of paraganglionic tumors, occurring in up to ~ 40 of adult cases and in up to 80% of pediatric ones. Patients with germline mutation often develop synchronous or metachronous multicentric tumors [102, 150, 152, 153].

Germline mutations mainly affect the following genes: *RET*, *NF1*, *VHL*, *TMEM127*, *SDHA*, *SDHB*, *SDHC*, *SDHD*, *SDHAF2*, *FH*, *MAX*, *EPAS1*, *DLST*, *MDH2*, *GOT2*, and *DNMT3A* (Table 1).

SDH (succinate dehydrogenase, complex II of the mitochondrial respiratory chain) consists of four subunits (SDHA, SDHB, SDHC, SDHD), and the genes encoding *SDHB* and *SDHD* are those more frequently mutated in the germline [153]. *SDHD* mutations cause the majority of paragangliomas of the head and neck region and typically present with single or multifocal tumors exclusively located in the head and neck. On the other hand, thoracoabdominal tumors more frequently harbor *SDHB* mutations. Paraganglionic tumors can also be found in association with gastrointestinal stromal tumors (GIST) and pulmonary chondromas in the so-called Carney triad, a nonhereditary condition characterized by epigenetic alterations of *SDHC* [154, 155]. Interestingly, somatic *NF1*, *RET*, and *VHL* are also found in sporadic tumors, with *NF1* representing the gene most commonly mutated ( ~ 20% of cases) [156, 157]. Additional somatic mutations, not found in the germline, affect *HRAS*, *BRAF*, *SETD2*, *FGFR1*, *TP53*, *ATRX*, *ARNT*, *IDH1*, *H3F3A*, *MET*, and *CSDE1* [146].

Paraganglionic tumors have been divided into three molecular clusters that are also recognized by the TCGA [145]. Cluster 1 tumors have a response to hypoxia pathway dysregulation (pseudohypoxia) characterized by increased transcription of genes targeted by hypoxia-inducible factors (HIF1-alpha and other factors) which promote angiogenesis, cell proliferation, survival, and epithelial-mesenchymal transition [158]. Of note, in *SDH*-/*FH*-deficient and mutant *IDH* tumors, oncometabolite accumulation induces DNA hypermethylation and other epigenetic changes. Cluster 2 tumors feature abnormal activation of RAS/RAF/ERK, PI3K/PTEN/AKT, and MYC/MAX/MXD1 pathways. They also exhibit a hypomethylated phenotype and frequent somatic copy number changes [146]. Cluster 3 tumors are characterized by dysregulation of Wnt and Sonic Hedgehog pathways. Indeed, sporadic *MAML3* fusions and *CSDE1* mutations in paraganglionic tumors that activate Wnt and Sonic Hedgehog pathways have been discovered to be major driving factors in tumor development [145]. Given the high hereditability of paraganglionic tumors, genetic screening is recommended, particularly in pediatric patients [150, 159]. In this respect, immunohistochemistry is a very useful screening test: if any of the subunits of the SDH complex is lost due to mutations or epigenetic alterations, the entire complex becomes unstable and the SDHB subunit is degraded in the cytoplasm. Loss of the SDHB protein can be demonstrated by SDHB immunohistochemistry, pointing to the need for SDH sequencing to confirm SDH subunit germline mutation [38]. This type of so-called “molecular-immunohistochemistry” can be applied not only to anticipate the genetic background of individual paraganglioma tumors but also to prevent erroneous diagnostic conclusions in the case of multiple lesions mimicking metastatic disease [146, 147].
