# Correction to: Molecular pathology of endocrine gland tumors: genetic alterations and clinicopathologic relevance

**DOI:** 10.1007/s00428-024-03738-3

**Published:** 2024-01-26

**Authors:** Antonio De Leo, Martina Ruscelli, Thais Maloberti, Sara Coluccelli, Andrea Repaci, Dario de Biase, Giovanni Tallini

**Affiliations:** 1 Department of Medical and Surgical Sciences (DIMEC), University of Bologna, 40138 Bologna, Italy; 2 Solid Tumor Molecular Pathology Laboratory, IRCCS Azienda Ospedaliero-Universitaria di Bologna, 40138 Bologna, Italy; 3 Division of Endocrinology and Diabetes Prevention and Care, IRCCS Azienda Ospedaliero-Universitaria di Bologna, 40138 Bologna, Italy; 4 Department of Pharmacy and Biotechnology (FaBit), University of Bologna, 40126 Bologna, Italy

**Correction to: Virchows Archiv**

10.1007/s00428-023-03713-4

In this article the keywords were incomplete. The complete keywords are presented as follows:

Keywords Endocrine gland tumors · Molecular pathology · Pituitary adenoma · PitNET · Thyroid tumors · Parathyroid tumors · Adrenal cortical tumors · Paraganglionic tumors

The text "BRAF V600E " should have been "BRAF p.V600E"

The original article has been corrected.

